# Multidomain correlates of burnout: A population-based study using supervised machine learning

**DOI:** 10.1007/s00127-026-03113-4

**Published:** 2026-05-26

**Authors:** Anja Monstadt, Yvonne Friedrich, Fabian Rottstädt, Mona Weiss, Thomas Behrens, Swaantje Casjens, Jonas Frost, Janka Massag, Irene Moor, Nils Opel, Laura R. Pfrommer, Benjamin Schüz, André Scherag, Martin Walter, Rafael Mikolajczyk, Ilona Croy

**Affiliations:** 1 Department of Clinical Psychology, Friedrich-Schiller-Universität Jena, Jena, Germany; 2 Partner Site Halle-Jena-Magdeburg, German Centre for Mental Health (DZPG), Jena, Germany; 3 Department of Work and Organizational Psychology, Friedrich- Schiller-Universität Jena, Jena, Germany; 4 Institute for Prevention and Occupational Medicine of the German Social Accident Insurance, Institute of the Ruhr University Bochum (IPA), Bochum, Germany; 5 Institute for Medical Epidemiology, Biometry, and Informatics, Interdisciplinary Centre for Health Sciences, Medical Faculty of the Martin, Luther University Halle-Wittenberg, Halle an der Saale, Germany; 6 Institute for Medical Sociology, Interdisciplinary Centre for Health Sciences, Medical Faculty of the Martin, Luther University Halle-Wittenberg, Halle an der Saale, Germany; 7 Department of Psychiatry and Psychotherapy, Charité – Universitätsmedizin Berlin, Berlin, Germany; 8 Institute of Public Health and Nursing Research, Universität Bremen, Bremen, Germany; 9 Institute of Medical Statistics, Computer and Data Sciences (IMSID), Universitätsklinikum Jena, Jena, Germany; 10 Department of Psychiatry and Psychotherapy, Universitätsklinikum Jena, Jena, Germany; 11 Department of Psychotherapy and Psychosomatic Medicine, University Hospital Carl Gustav Carus, Technische Universität Dresden, Dresden, Germany

**Keywords:** Burnout, Supervised Machine Learning, Mental Health, Organisational Psychology

## Abstract

**Purpose:**

Burnout is a growing challenge for individuals and society and understanding risk factors is crucial to develop targeted prevention strategies. Thus, we aimed to determine whether and how burnout relates to different organisational factors, psychosocial employment conditions, sociodemographic variables and mental health.

**Methods:**

We analysed cross-sectional survey data collected in the 2023 wave with focus on occupational health within the German population-based DigiHero cohort. Using linear associations and xg-boost machine learning, we explored the relations of multidomain variables with burnout severity, which was measured with the Maslach Burnout Inventory. Machine learning results were interpreted through SHAP values.

**Results:**

We included 27,020 participants, aged 18–67 years (median 49), 65.2% female, in the analysis. The strongest predictors of burnout were effort-reward-imbalance, work-life-interference, overcommitment and poor general mental health, particularly symptoms of depression and anxiety. Compared to psychosocial employment conditions and individual mental health, organisational and sociodemographic factors were less important. However, occupation mattered and other aspects, such as extended remote work, had small, but noticeable effects. Age and income were the only sociodemographic factors that contributed. Results from xg-boost were compared to results from univariate statistics and remained robust when adjusting for sample bias through case weights.

**Conclusion:**

The study emphasises the importance of psychosocial employment conditions and individual mental health in the context of burnout, which outweigh the effects of organisational and sociodemographic factors. For practice this suggests that positive social work environments may prevent burnout while mental health problems increase vulnerability. For causal insights, prospective longitudinal studies are required.

**Supplementary Information:**

The online version contains supplementary material available at https://doi.org/10.1007/s00127-026-03113-4.

Burnout is a clinical condition that develops when workplace stressors chronically exceed a person’s resources [1] and expresses in exhaustion, cynicism, and a reduced sense of effectiveness [2]. Far from being just an individual issue, burnout has become a growing challenge for society. In Germany for instance, 4.2% of people are estimated to experience clinical burnout at some point in their life [3] and work-incapacity days attributed to burnout have more than doubled in the last ten years [4]. This trend is particularly concerning in the light of demographic change with increasing pressure on social security systems.

For those affected, burnout has serious consequences. It is linked to a wide range of health problems, including musculoskeletal pain, and even increased mortality [5–7]. Comorbidity with mental disorders is especially high, affecting 70.9% of people with burnout [3]. This is partly based on a conceptual overlap between burnout and depression [8–11]. In the workplace, burnout is linked to job dissatisfaction, turnover intentions and absenteeism [7, 12]. Prospective data shows for instance, that higher levels of burnout predict significantly more sickness absence days: An increase of work-related burnout by one standard deviation at baseline predicted 21% more sickness absence days at follow-up [13].

Understanding the causes of burnout is essential to develop targeted prevention strategies. A widely used framework is the Job Demands-Resources Model [14], which explains burnout as a lack of sufficient resources to buffer chronically high job demands. High demands primarily contribute to exhaustion, while insufficient resources foster disengagement and cynicism.

In line with the predictions of the model, high ***organisational*** demands such as long working hours, high workload, time pressure, workplace insecurity, role conflicts, and working in care-giving professions are associated with a higher burnout risk – as are low resources such as lack of autonomy, insufficient salary, no possibility to influence important decisions, and little experience in the field [1, 15–18] (for meta-analysis see [19, 20]).

In addition to the Job Demands-Resources Model, Siegrist’s [21] model of Effort-Reward-Imbalance puts more emphasis on perceived rewards and posits that a mismatch between high investment and low gains at work increases the risk for stress-related disorders. Rewards include not only monetary aspects such as income, but also ***psychosocial*** rewards such as support, recognition and appreciation. Another relevant concept is work-life balance [22], which states that an interference between professional and private life demands causes stress. The importance of keeping effort-reward and work-life in balance is reflected in various risk factors of burnout, e.g. poor professional relationships, lack of recognition for quality improvement activities, challenges in work-life integration, low support from supervisors and co-workers, and workplace injustice [17, 19, 23]. For example, bullying at work and effort-reward-imbalance are the strongest predictors of burnout in health care professionals [18] and adverse work-life balance, as well as a negative workplace culture, are significant predictors of burnout in veterinarians [24].

Concerning ***sociodemographic factors***, the effect on burnout is inconsistent, small or non-significant [25, 26]. Gender for instance is associated to different burnout symptomatology, as revealed in a meta-analysis: Women score higher on emotional exhaustion while men have higher values on cynicism [27]. Some studies found that burnout decreases with age [28, 29], but a German population-based study in about 8,000 individuals conversely reported more frequent burnout in older people [3]. Furthermore, people with higher education and married people show lower levels of burnout [28].

Finally, ***mental health*** problems are not only a consequence of burnout, but also a risk factor. A longitudinal study in over 2,500 dentists shows that the risk of developing burnout increases 2.6-fold for people who had depressive symptoms at baseline [30]. Similarly, depression and burnout had reciprocal effects in young workers assessed over eight years after graduation [31]. In addition to depression, anxiety, general psychological distress, difficulties in emotional regulation, somatic symptoms, and general reduced well-being also predict burnout [32–35].

Taken together, organisational, psychosocial, sociodemographic factors and mental health contribute to burnout. Although these factors have been widely researched and integrated in reviews and meta-analyses [18–20], their relationships and relative importance remain unclear, as existing studies are often limited by a narrow set of predictors. For example, a large longitudinal study with a population-based sample of about 2,000 participants and a 3-year follow-up assessment included only eight psychological and somatic predictors of burnout [35]. Such restricted predictor sets hinder the weighing of the relative contribution of multiple factors, which in turn limits the prioritisation of prevention strategies. In addition, generalisability of previous findings is constrained by highly specific samples. Even larger studies with sample sizes in the range of 2,000–8,000 individuals, have frequently focused on single professions, such as veterinarians [24] or dentists [30].

To provide a more comprehensive picture of statistical predictors of burnout in the general population, we analysed data from a large German cohort study including a variety of potential predictors spanning multiple domains. This enabled us to employ supervised machine learning, a method suited to handle large data sets with interdependent predictors and capable of detecting non-linear patterns and interactions. Our aim was to identify the most important predictors of burnouts and estimate the magnitude of effects and particular feature contributions with respect to potential non-linear patterns and interdependencies.

## Methods

### Recruitment

Participants were surveyed within the German cohort study for digital health research (DigiHero [36]; compare Online Resource for more information on recruitment strategy), which is a digital platform assessing health related factors in Germany. The sample size was 90,842 participants at the time of the collection wave focussing on occupational health, which we used for the current analysis (November 2023).

## Measures

***Burnout*** was measured with the Maslach Burnout Inventory – General Survey (MBI [37]). It consists of 16 items answered on a 7-point fully anchored scale ranging from 0 (*“never”*) to 6 (*“daily”*) which were averaged for the overall score and three subscales: emotional exhaustion, cynicism (also referred to as depersonalization) and personal efficacy (also referred to as professional achievement). To ease reading, we recoded the latter to “reduced personal efficacy” so that higher values in each scale represent higher strain.

***Organisational factors*** were queried in form of occupation (free text item), professional field (according to the German classification of economic sectors [38]), occupational area, main group and group (according to the German classification of occupations up to the third level [39]), contractually agreed and actual weekly working hours (continuous), hours spent working from home (remote work; continuous), leadership position (y/n), employment duration (continuous) and type (employed with permanent contract, employed with fixed-term contract, civil servant, self-employed, temporary work), personal contact with clients (4-point ordinal scale), worktime at (computer) screen (4-point ordinal scale), shift work (y/n), having more than one employment (y/n), and working overtime (y/n).

***Psychosocial employment conditions*** were measured with the Effort-Reward-Imbalance Scale (ERI [40]) containing 16 items, of which 10 measure effort-reward-imbalance and 6 overcommitment. The 4-point Likert scale ranges from 1 (*“totally disagree”*) to 4 (*“totally agree”*). Subjective interference of work and private life was measured with a single item on a 5-point scale ranging from 1 (*“to a very small degree”*) to 5 (*“to a very large degree”*).

***Sociodemographic variables*** were retrieved from baseline data collection and included age, gender, marital status, partnership, education, school leaving qualification, monthly household net income (7-point ordinal scale; for brevity hereafter referred to as income), state and federal district, number of household members, origin of participant and their parents, age of arrival in Germany, number of their own children in household.

***Mental health*** was assessed through different constructs. Depression was measured with the Patient Health Questionnaire-9 (PHQ-9 [41]), anxiety with the Generalized Anxiety Disorder Scale-7 (GAD-7 [42]), and (psychosomatic) pain with the items b, c and e of the Patient Health Questionnaire 15-Item Somatic Symptom Severity Scale (PHQ-15 [43]). Life satisfaction was averaged from three items, asking how satisfied, meaningful, and happy participants perceive their lives on an 11-point scale ranging from 0 (*“Not at all satisfied/meaningful/happy”*) to 10 (*“Very satisfied/meaningful/happy”*). Resilience was measured with the items 1 and 8 of the Connor Davidson Resilience Scale (CD-RISC [44]) and subjective mental health with a single item on a 5-point scale ranging from 1 (*“Bad”*) to 5 (*“Excellent”*).

## Analysis strategy

All statistical analyses were computed using R and RStudio [45]. Data preparation was realised with the tidyverse [46], which includes ggplot2 [47] used for data visualization. Patchwork [48] was used additionally to arrange multiple plots. Machine Learning Models were calculated with tidymodels [49] and SHapley Additive exPlanation (SHAP) values were computed with shapviz [50].

## Preprocessing

Questionnaire means were used if missing values did not exceed 20%. Internal consistency was computed for each questionnaire that comprised at least three items, using Cronbach’s α [51]. The variables education, school leaving qualification, and monthly household net income were transformed into numeric scales prior to analysis. The free-text item occupation was recoded to higher-level categories, e.g. “teacher” instead of “high school teacher for maths and music” and corrected for typing errors and spelling mistakes by semi-automated analysis. Occupations with less than 10 mentions were summarized as “other”, which affected 14.3% of the participants.

## Univariate statistics

We assessed the associations with burnout using Pearson correlations for numeric variables, point-biserial correlations for dichotomous variables and simple linear regressions for factor variables, which equals an analysis of variance (ANOVA). To investigate the association to occupation and occupational group, the mean burnout scores were calculated per category (if *n* ≥ 30 participants). To compare the effect sizes of each variable, the adjusted R² was calculated. According to Cohen [52], the magnitude of the effects was classified as follows: a small effect starting from R² = 0.01, medium effect from R² = 0.09 and a large effect from R² = 0.25. We only report variables that had at least a small effect.

### Machine learning

We used supervised machine learning regression models to detect key features associated with burnout severity and potential non-linear patterns and interactions. Machine learning is a broad term that includes a series of very flexible, but computationally intensive, statistical models such as decision tree ensembles and artificial neural networks. In contrast to traditional statistical methods, such machine learning models operate without statistical assumptions or mathematical definition of the relationship between features (predictors) and outcome (here burnout), hence they can deal with non-linear patterns and complex interactions.

**Choice of Machine Learning Algorithms.** We used an exploratory approach: multiple machine learning models were fitted to the training set (80%) using 10-fold cross-validation to tune models via grid search [53] and their performance was evaluated on the test set (20%). The splitting was performed using the overall burnout mean for stratification. The best tuning parameters for each model and the model performing best overall were chosen based on the root mean squared error (rmse). As visual data inspection revealed mostly linear relationships, we applied a linear regression model and additionally linear models that include shrinking/eliminating coefficients (ridge, lasso and elastic net). Other machine learning algorithms were selected based on their prior performance in other studies: Tree ensembles such as random forest and xg-boost (eXtreme Gradient Boosting) have become popular for their excellent performance in various applications [54–57] and we used multilayer perceptron models as a simple form of artificial neural networks [58], which have often performed well [55, 57].

**Preprocessing within the Machine Learning Framework.** The recipes package used in the workflow from the tidymodels framework automatically performs preprocessing on the training data and applies the same parameters to the test set, which guarantees there is no data leakage. Preprocessing steps were added sequentially: First, missing values for all features were imputed with the k-nearest-neighbour algorithm, which previously performed well [59]. Thereafter, factor variables were treated, depending on the requirements of the specific machine learning algorithm. For every model, very rare categories (< 10 observations) were pooled to an “other” category. Subsequently, high cardinality factors (> 15 levels) were transformed into likelihood encodings via step_lencode_glm(). For linear (lm, lasso, ridge, elastic net) and multilayer perceptron models other factor variables were dummy coded, for random forest no encoding is necessary, and for xg-boost one-hot encoding was used. For multilayer perceptron models, all features were additionally z-standardised to be on the same scale.

**Model Selection.** The xg-boost and multilayer perceptron model performed best (compare eTable 1 in the Online Resource), although all models reached similar performance levels according to rmse. We decided to continue with the xg-boost model as it allows the calculation of SHAP values which ease interpretation [60]. The inspection of likelihood encodings revealed no association between burnout and district, therefore this variable was excluded from further analysis. To adjust for sample bias of gender, age and school leaving qualification, we reran the model with case weights (compare Online Resource for detailed description).

**Model Interpretation.** Variable importance scores were computed with the default method from the vip package [61] which is the fractional contribution of each feature to the model based on the total gain for xg-boost models. SHAP values were computed, which represent the feature contribution to the estimated outcome for each person [62]. We also computed SHAP interaction values. We restricted the SHAP value calculation to a random subset of 2,000 participants due to high computation time.

## Results

### Sample descriptive statistics

We included 27,020 participants aged 18–67 years (*M* = 46.8 years, *SD* = 11.16 years; 65.2% female; Table 1). The original sample size of 90,842 participants was reduced to those who answered to the wave of data collection with focus on occupational health (47,640), were currently working (29,189), were younger than retirement age (67 years; 28,499), had no more than 3 missing values in the MBI [37] (27,174), and submitted plausible values for employment duration (no longer than age minus 14 years; 27,020). Internal consistency measures (Cronbach’s α) for questionnaires were close to 0.80 or above (burnout total = 0.900, emotional exhaustion = 0.922, cynicism = 0.872, reduced personal efficacy = 0.829, depression = 0.846, anxiety = 0.869, effort-reward-imbalance = 0.786, overcommitment = 0.822, life satisfaction = 0.906), apart from the items on pain (0.544).

**Table 1:** Sample Description

Variable	Category	*N*	%
Gender	Male	9308	34.4
	Female	17,629	65.2
	Other	34	0.1
	*NA*	49	0.2
Education	PhD (4)	1389	5.1
	Master (3)	9596	35.5
	Bachelor (2)	1636	6.1
	Vocational Training (1)	13,385	49.5
	None (0)	675	2.5
	*NA*	339	1.3
School Leaving Qualification	Degree after 12th or 13th grade (Abitur; 3)	17,894	66.2
	Degree after 10th grade (Realschulabschluss; 2)	8229	30.5
	Degree after 9th grade (Hauptschulabschluss; 1)	556	2.1
	None (0)	64	0.2
	*NA*	277	1.0
Monthly Household Net Income	5000€ or more (7)	6443	23.8
	4000€ up to less than 5000€ (6)	5210	19.3
	3000€ up to less than 4000€ (5)	5402	20.0
	2250€ up to less than 3000€ (4)	3761	13.9
	1750€ up to less than 2250€ (3)	2152	8.0
	1250€ up to less than 1750€ (2)	1383	5.1
	Less than 1250€ (1)	630	2.3
	*NA*	2039	7.5
Country of Birth	Germany (within present borders)	26,327	97.4
	Other Country in Europe	453	1.7
	Country Outside of Europe	196	0.7
	*NA*	44	0.2
Marital Status	Married	16,301	60.3
	Not Married	7318	27.1
	Divorced	2199	8.1
	Widowed	410	1.5
	Married, but Separated from Partner	456	1.7
	*NA*	336	1.2
Partnership	Yes	21,685	80.3
	No	4997	18.5
	*NA*	338	1.3

*Variables [Questionnaire]*	*N Items*	*Mean*	*Median*	*SD*	*Range*	*NA*
Age	1	46.798	49.000	11.160	18–67	0 (0%)
Contractually agreed weekly working hours	1	34.617	38.500	8.427	0–60	404 (1.5%)
Actual weekly working hours	1	38.146	40.000	9.866	0–80	245 (0.9%)
Burnout [MBI]	16	1.740	1.562	1.028	0–6	0 (0%)
- Emotional Exhaustion	5	2.432	2.200	1.518	0–6	5 (< 0.1%)
- Cynicism	5	1.459	1.000	1.398	0–6	35 (0.1%)
- Reduced Personal Efficacy	6	1.396	1.167	1.050	0–6	114 (1.0%)
Depression [PHQ-9]	9	0.674	0.556	0.503	0–3	384 (1.4%)
Anxiety [GAD-7]	7	0.824	0.714	0.604	0–3	365 (1.4%)
Effort-Reward-Imbalance [ERI]	10	2.547	2.500	0.511	1–4	1399 (5.2%)
Overcommitment [ERI]	6	2.493	2.500	0.639	1–4	510 (1.9%)
Life Satisfaction	3	7.115	7.667	1.733	0–10	396 (1.5%)
Resilience [CD-RISC]	2	3.151	3.000	0.695	0–4	370 (1.4%)
Pain [PHQ-15]	3	0.684	0.667	0.508	0–2	537 (2.0%)

Note. Descriptive statistics of final sample of 27,020 participants for the most important features. Factor variables are displayed in the upper part and questionnaires and continuous variables in the lower part. N = number (count), NA = missing values, SD = Standard Deviation, descriptive information of all variables can be found in eTable 2 in the Online Resource

**Fig. 1:**
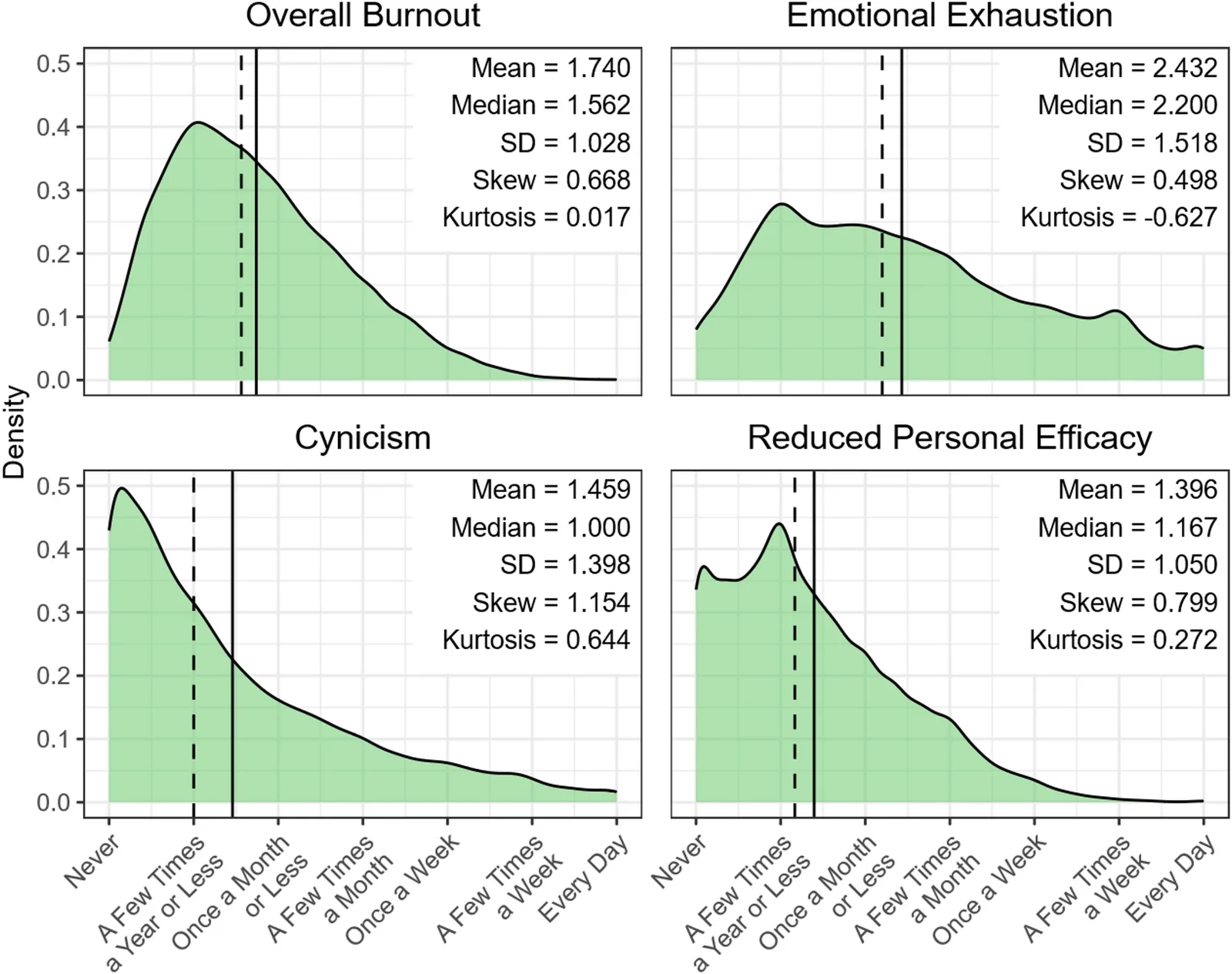
Distribution in the final sample of 27,020 participants of the overall mean of the Maslach-Burnout-Inventory (16 item mean), and its three subscales emotional exhaustion (5 item mean), cynicism (5 item mean) and reduced personal efficacy (6 item mean). The plain line represents the mean, the dashed line represents the median. SD = standard deviation

Most people (63.1%) reported that burnout symptoms occur once a month or less frequently and very few respondents (2.6%) stated experiencing symptoms weekly or more often. Among the subscales, emotional exhaustion had the highest proportion of elevated scores. In contrast, cynicism scores were generally lower and for reduced personal efficacy, very high values were very rare (Fig. 1).

### Univariate Linear Associations

Organisational factors showed weak relations to burnout (Table 2). Being in a leadership position and self-employment were associated with lower burnout. Furthermore, occupation and occupational group mattered with for instance teaching, metal processing and public relations occupations exhibiting higher burnout whereas woodworking and processing, and personal care occupations as well as management and executive board positions show lower burnout (eFigures 1 and 2 and eTables 4 and 5 in the Online Resource). In the area of psychosocial employment conditions, burnout was strongly associated with effort-reward-imbalance, and moderately with overcommitment and work-life-interference. None of the sociodemographic factors showed a meaningful relation to burnout. Concerning mental health, there was a strong correlation with depression, anxiety, reduced life satisfaction and reduced subjective mental health and a medium correlation with reduced resilience and higher pain. As subscales were highly correlated to the overall score, we focused the machine learning analysis on the overall score.

**Table 2:** Simple Linear Associations with Burnout and its Subscales

Variable	*R*² Overall	Cor/β Overall	Cor/β CY	Cor/β EE	Cor/β PE
Burnout: Cynicism	0.751	0.867	1.000	0.630	0.394
Burnout: Emotional Exhaustion	0.673	0.820	0.630	1.000	0.235
Burnout: Reduced Personal Efficacy	0.434	0.659	0.394	0.235	1.000
Depression	0.407	0.638	0.522	0.638	0.315
Anxiety	0.311	0.558	0.444	0.589	0.253
Life Satisfaction	0.306	−0.553	−0.457	−0.473	−0.368
Subjective Mental Health	0.295	−0.543	−0.409	−0.521	−0.335
Effort-Reward-Imbalance	0.274	0.524	0.477	0.533	0.193
Overcommitment	0.215	0.464	0.318	0.595	0.137
Work-Life-Interference	0.202	0.450	0.355	0.526	0.145
Resilience	0.157	−0.396	−0.282	−0.311	−0.347
Pain	0.095	0.308	0.227	0.381	0.091
Occupation*	0.022	*NA*	*NA*	*NA*	*NA*
Employment Type*	0.015	*NA*	*NA*	*NA*	*NA*
- Employed with Fixed-Term Contract	*NA*	0.127 0.076 to 0.178	0.037 −0.032 to –0.106	0.076 0.001 to 0.151	0.245 0.194 to 0.297
- Civil Servant	*NA*	0.114 0.072 to 0.156	0.055 −0.002 to 0.113	0.114 0.052 to 0.176	0.160 0.117 to 0.203
- Temporary Work	*NA*	0.234 −0.014 to 0.483	0.230 −0.114 to 0.574	0.055 −0.314 to 0.423	0.386 0.132 to 0.640
- Self-Employment	*NA*	−0.464 −0.515 to −0.414	−0.585 −0.655 to −0.515	−0.465 −0.540 to −0.389	−0.373 −0.425 to −0.321
Leadership Position	0.012	−0.109	−0.108	−0.029	−0.129
Professional Group*	0.011	*NA*	*NA*	*NA*	*NA*

Note. Simple linear associations with burnout mean and subscales, ordered by R². Cor = Correlation, β = regression coefficient, Overall = Overall Burnout, EE = Emotional Exhaustion, CY = Cynicism, PE = Reduced Personal Efficacy, NA = not applicable. For factor variables marked with an asterisk (*), correlations are not applicable. Categories from employment type are displayed with regression parameters (β). 95% confidence intervals are provided for β parameters as estimate certainty depends on subgroup size, while all other effects are significant due to the very large sample. Reference level for employment type = Employed with permanent contract. Variables with R² < 0.01 can be found in eTable 3 in the Online Resource. Visualisation for the associations of burnout with different occupations and professional sectors can be found in the Online Resource

### Burnout prediction with XG-Boost

**Fig. 2:**
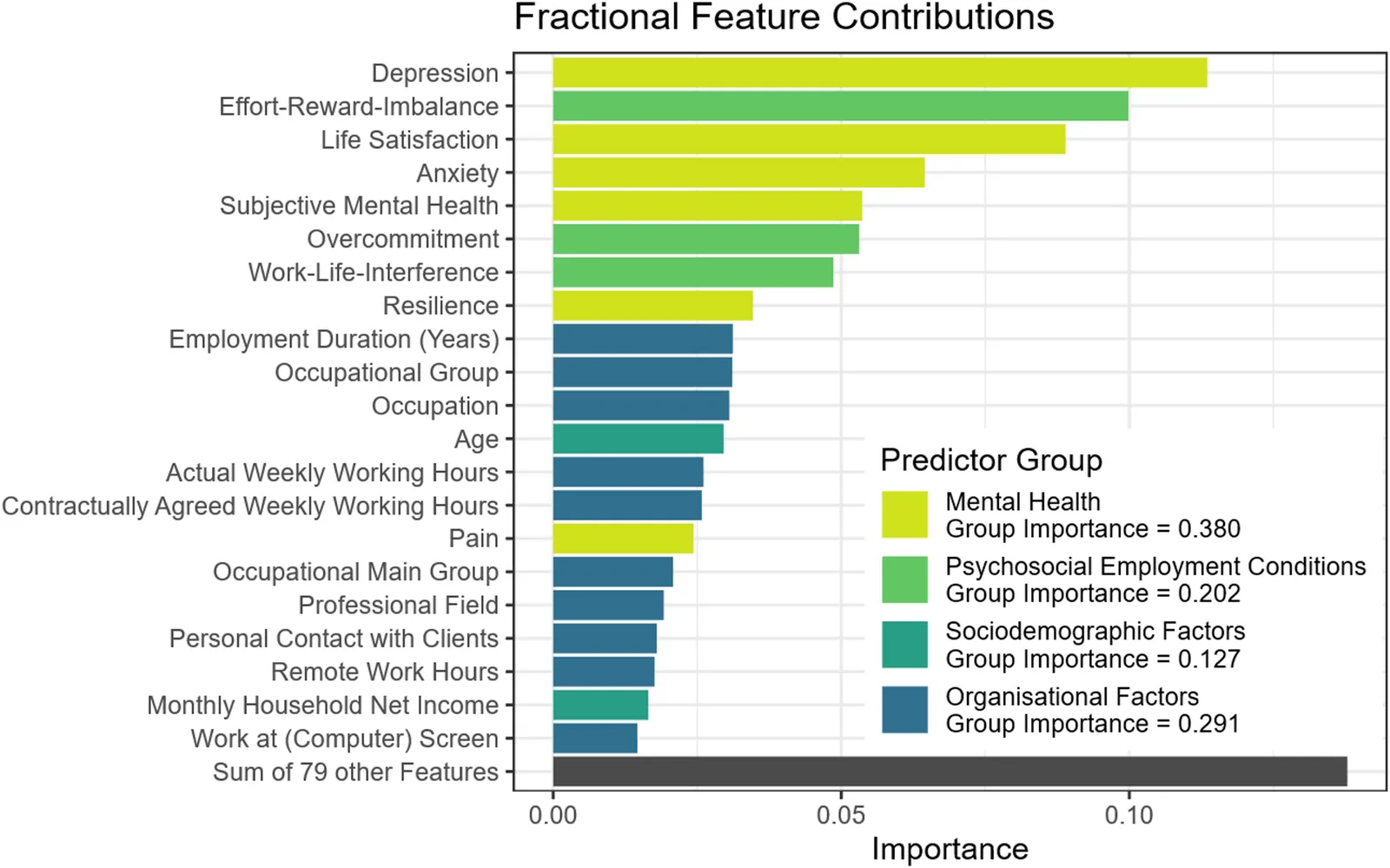
Variable importance scores from xg-boost model of features of burnout, features are sorted by importance. Feature groups are colour coded, the group importance is the sum of fractional feature contributions of each variable in this group. High group importance can also occur due to many features with low importance in one group

**Fig. 3:**
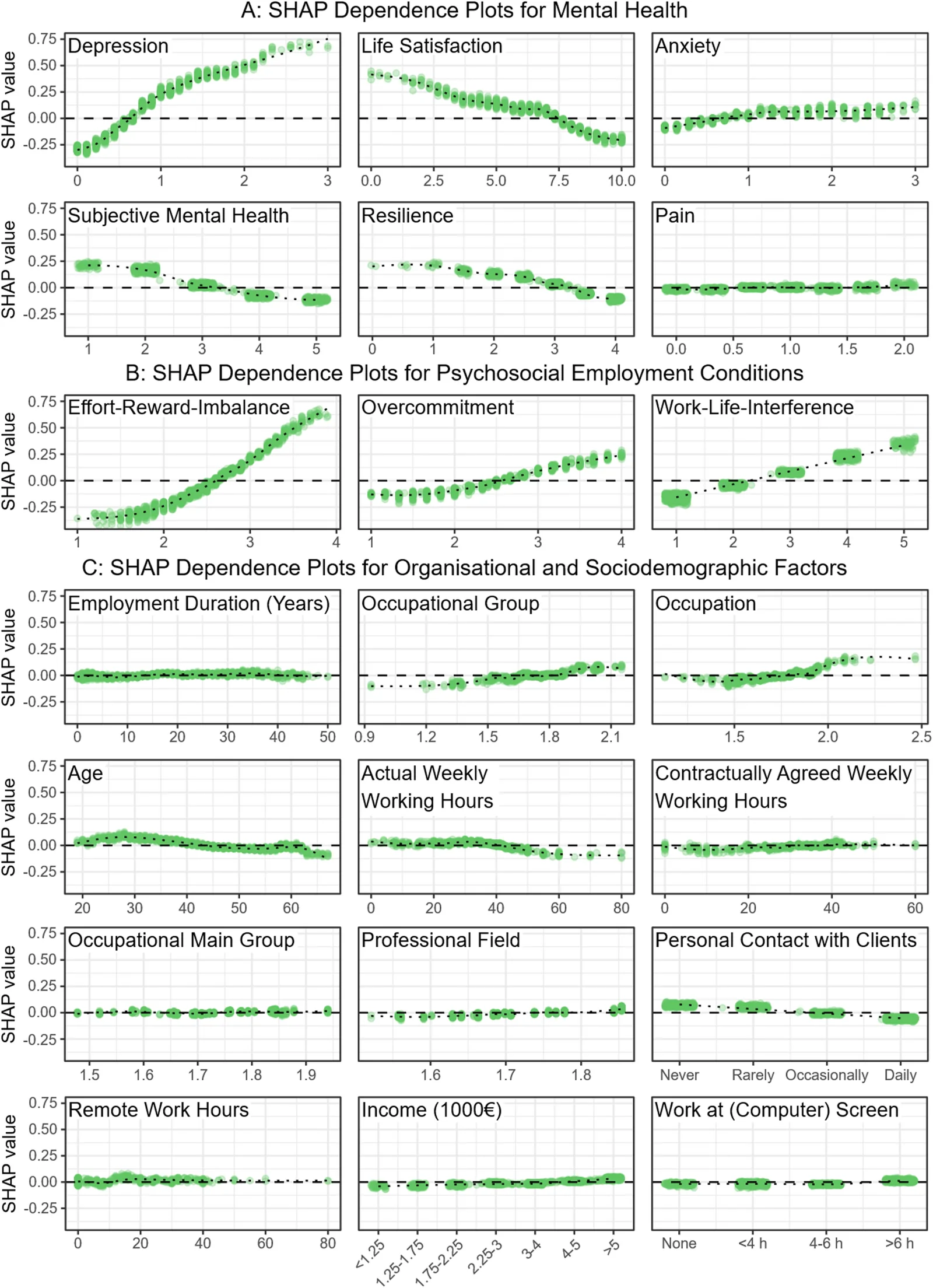
SHAP dependence plots for the most important features of burnout according to their fractional feature contributions. SHAP values represent the contribution of this feature to the estimated outcome for each person. A negative SHAP value means that the associated feature lowers the outcome (= lower burnout), positive SHAP values mean that it increases the outcome (= higher burnout). Greater absolute SHAP values mean stronger influence on the outcome. Feature values are represented on the x-axis. Ordered by feature group: A = mental health variables, B = psychosocial employment conditions and C = sociodemographic and organisational factors. Ordered by variable importance within each cluster. For features with less than ten values, points are randomly jittered on the x-axis to better visualise the amount of points. In the Online Resource, eFigure 5 provides plots with individual y-axis scales to better visualize small effects; eFigures 1 and 2 provide the associations of different occupations and occupational groups with burnout to interpret the likelihood encodings

To analyse all variables jointly we used the xg-boost model, which reached an R² = 0.592 and showed 21 features that stood out as most important. We focused on those as visual inspection revealed a drop in variable importance thereafter (Fig. 2). As in univariate statistical analysis, burnout was primarily related to mental health and psychosocial employment conditions, whereas organisational and sociodemographic variables were of comparably lesser importance (Fig. 2 and eFigure 3 in the Online Resource). The latter two had a relatively high importance sum, due to the high number of features which individually were not important. Machine learning also revealed some associations that differed from univariate analysis. For instance, working hours were among the most important features in the xg-boost model, but had only negligible effects in the univariate analysis, while it was the other way around for employment type and being in a leadership position. Finally, we closely examined the associative pattern of the first 21 features. SHAP values indicated many close to linear relationships to burnout, but also some exceptions (Fig. 3 and eFigs. 4 and 5 in the Online Resource). SHAP interaction values were inspected but turned out to be negligible.

***Mental health*** showed strong associations with burnout. Burnout increased nearly linearly with higher levels of depression and lower levels of life satisfaction, subjective mental health, and resilience (Fig. 3A). Higher anxiety was also related to burnout, although the association was weaker and plateaued early. Compared to the other mental health variables, the relation with pain was neglectable. Concerning ***psychosocial employment conditions***, effort-reward-imbalance, overcommitment and work-life-interference all strongly related to burnout positively in a nearly linear manner (Fig. 3B). Age and income were the only relevant ***sociodemographic factors*** (Fig. 3C). Burnout increased until the age of the late twenties, declined steadily thereafter, and showed a second raise around age 60. Burnout increased with higher income. For ***organisational factors***, occupation, occupational main group, group, and field were important with small effects (Fig. 3C and eFigs. 1 and 2 in the Online Resource; detailed dependence plots with adjusted y-axis scales are provided in eFigure 5 in the Online Resource). Results from likelihood encodings need to be interpreted carefully, as they were overestimated for occupation in the extremes and for the occupational main group underestimated in the medium values. In addition, higher burnout related to less personal contact with clients and medium employment durations (~ 15–35 years). Burnout was lower in persons who worked more than ~ 35 h, though a contractually agreed weekly working time of ~ 10–30 h was also associated with lower burnout. Remote work related to increased burnout only when exceeding ~ 10 h per week and screen work only when exceeding six hours daily. The ***weighted model*** reached an R² = 0.589 and revealed comparable results, including the same top 21 features (compare Online Resource).

## Discussion

Our large sample supported and extended previously described factors relating to burnout. Our analysis strategy was twofold. First, we studied univariate associations between predictors and burnout using conventional statistics. This supported previous results, for instance showing that effort-reward-imbalance shows a strong association with burnout [14]. Second, we employed machine learning which enabled us to weigh the relative influence of different factors, and account for their interdependency and non-linear association patterns. Machine learning results were largely comparable with conventional statistics but also showed that some associations were altered when considering the interdependency of features. For instance, being in a leadership position was strongly associated with burnout in the univariate analysis but showed only negligible effects in the machine learning analysis. This indicates that the isolated examination of predictors is not suitable to understand the pattern of associations. In the next sections, we will discuss the machine learning results in detail.

We found that ***mental health***, particularly depression, had the largest influence on burnout, which is unsurprising given the conceptual overlap between burnout and depression [8]. However, our study also points to a general mental health factor influencing the vulnerability to burnout, as anxiety, lower subjective mental health, reduced life satisfaction, and diminished resilience were likewise related.

Supporting the effort-reward-imbalance model [21], ***psychosocial factors of employment*** were highly important. Effort-reward-imbalance is strongly associated with burnout, consistent with previous research which showed that this construct is also a risk factor for depression and mental disorders more broadly [63, 64]. Besides that, overcommitment and work-life-interference related to burnout, even when controlling for mental health. Given the large sample spanning many occupations and sociodemographic groups, these factors appear of general importance. Preventive strategies should therefore prioritize psychosocial aspects, for example by enhancing rewards through recognition, supporting professional development and career management [21], and promoting flexibility and autonomy, such as providing remote work options – while maintaining regular social contact to prevent isolation.

***Sociodemographic variables*** had comparably little influence. Consistent with prior studies, burnout decreased with age [28, 29], despite a slight rise until the late twenties. This early peak may reflect the greater mental health vulnerability in younger adults [65], due to the combined demands of learning new job tasks and life-stage challenges such as starting a family.

***Organisational factors***, such as short employment duration and occupation were also of minor importance. Employment duration likely reflects the effect of work experience [66]. Consistent with the original focus of burnout research on people-oriented, human service occupations, our study showed that occupations such as teaching occupations were at higher risk. However, in line with a broader conceptualisation of burnout [67], we also demonstrate that burnout is not limited to helping professions. Working hours were seemingly contradictory: Both part-time employment and very long actual working hours appeared protective. This is likely explained by different mechanisms: Part-time work facilitates the work-life balance, whereas working more than 40 h a week may reflect high intrinsic motivation. More client contact and lesser screen-based work were associated with lower burnout, supporting job embeddedness theory [68], which highlights the role of social ties. In a similar way, extensive remote work (> 10 h per week) was adverse, whereas moderate remote work appeared beneficial. Surprisingly, higher income related to slightly higher burnout, though this effect was small and may reflect measurement limitations in the income, which provided a finer resolution in lower income.

### Limitations

First and foremost, interpretation is limited by the cross-sectional data, hence the identified predictors may be antecedents or consequences of burnout, or a relationship may occur because of an underlying third variable influencing both the feature and outcome. Some variables have also been identified in prospective studies [19] and align with theoretical assumptions [14, 21], supporting their predictive relevance. However, other directionality is also plausible, particularly for mental health. For example, an overloaded individual may withdraw from social activities or develop a cynic perspective, both of which can foster depressive symptoms, where the reduction of energy can in turn aggravate the feeling of being overwhelmed by workload. Longitudinal studies support such reciprocal relationships [30, 31]. Second, the sample is not fully representative of the general population, despite being randomly drawn from population registers. Nonresponse led to an overrepresentation of female, 40–60-year-old, and well-educated individuals. We addressed this through case weight modelling, which supports generalisability for these factors. Nevertheless, individuals with migration background remained underrepresented and thus our results are best applicable to WEIRD (western, educated, industrialised, rich, democratic) populations [69]. Third, machine learning models are complex which hinders interpretability. Though SHAP values provide an approach to inspect the associations, they do not capture complex higher-level interactions, which may exist in the real world. Fourth, while our set of predictors was broad, it was limited by the survey design. Other potentially important factors, such as personality traits [70], or quality of work-related relationships [71] were not assessed, and some variables (screen time, income), were simplified through ordinal scaling, with income categories offering no resolution above the national average. Our results are only as specific as the data and not comprehensive for all potential factors influencing burnout. Finally, the MBI, though widely used in research, lacks standardised cut-offs for clinical burnout [72], we thus cannot draw any conclusion on the prevalence. Also, our sample likely underrepresents severe cases, as it only included employed participants. Taken together, future research should include prospective longitudinal designs and diverse samples with broad predictor sets to gain causal insights. Our results may inform the choice of predictors for confirmatory studies.

### Practical Implications

Our data indicates that psychosocial variables should be prioritized in prevention efforts, as they show the strongest association to burnout and can be addressed at the organisational level. For example, management training programs may emphasize aspects of workplace security, appropriate appreciation, adequate promotion opportunities, as described in the effort-reward-imbalance model. Furthermore, granting flexible working conditions through remote work arrangements may reduce burnout risk but requires balancing flexibility with maintaining social connection. In addition, work-life balance and income related to burnout, pointing to broader structural conditions relevant for prevention. Finally, individual factors including poor mental health, young age and employment in certain professions, showed associations to elevated burnout. Prevention may therefore especially target those individuals to support mental health at work.

## Conclusion

This study used a large dataset and machine learning to identify predictors of burnout. Mental health and psychosocial factors emerged as the most important statistical predictors, while organisational and sociodemographic factors had a smaller influence. For causal insights, prospective longitudinal studies are required. 

. 

## Electronic Supplementary Material

Below is the link to the electronic supplementary material.

Supplementary Material 1

## Data Availability

Data from the DigiHero study is not publicly available due to participant privacy considerations and ethical restrictions that prevent open sharing. Access may be granted to qualified researchers upon reasonable request and after approval from the DigiHero Use and Access Committee.
