# Supplementary material for: Multidomain correlates of burnout: A population-based study using supervised machine learning
Source: Soc Psychiatry Psychiatr Epidemiol. 2026 May 26;61(11):2435–47. doi: 10.1007/s00127-026-03113-4 (PMC13694440; doi:10.1007/s00127-026-03113-4)
Supplement: Supplementary file 1 [file 127_2026_3113_MOESM1_ESM.pdf]

Supplement to the Article "Multidomain Correlates of Burnout: A Population-based Study Using Supervised Machine Learning"

Table of Contents

DigiHero recruitment strategy 3

Results from Model Tuning 4

eTable 1: Results from Model Tuning and Test Set rmse and R^2 4

Full Variable List 5

eTable 2: Sample Descriptive Statistics for all Variables 5

Effect Sizes for all Variables 8

eTable 3: Simple Linear Associations with Burnout 8

Association of Burnout with Occupation and Occupational Group 11

eFigure 1: Wordcloud for the Association of Burnout with Occupation 11

eFigure 2: Wordcloud for the Association of Burnout with Occupational Group 12

eTable 4: Mean Burnout Values by Occupation 13

eTable 5: Mean Burnout Values by Occupational Groups 17

Fractional Feature Contributions for all Variables 22

eFigure 3: Variable Importance Plot 22

Beeswarm Plot for the 21 Most Important Features 23

eFigure 4: Beeswarm Plot 23

SHAP Dependence Plots with Individual Y-Axis Scales 24

eFigure 5: SHAP Dependence Plots 24

Model with Case Weights 26

Method 26

Results 26

Limitations 26

eFigure 6: Variable Importance Plot for Weighted Model 27

eFigure 7: Beeswarm Plot for Weighted Model 28

eFigure 8: SHAP Dependence Plots for Weighted Model 29

References 31

DigiHero recruitment strategy

Participants were surveyed within the DigiHero study [1], which is a digital platform assessing health related factors in Germany with a sample size of 90,842 participants at the time of the survey of the current analysis (November 2023) and still recruiting. Recruitment is population based, with random samples driven from the population registers of selected regions (all districts in the federal states of middle Germany (Saxony, Saxony-Anhalt, and Thuringia) and selected districts in other federal states. In those districts around 30% of the population were invited, in cities > 100,000 inhabitants restricting at 30,000 invitees. The mean response proportion is at 3%, varying between 2-8% across different regions. After the online registration, the participation in the study is fully online with 3-4 questionnaires per year, covering different topics. Sociodemographic aspects are assessed in a baseline survey when participants join the DigiHero cohort. The November 2023 survey on occupational health was sent out to all participants registered at the time and was answered by 47,640 (52.4%) persons. In our analysis, we included data from participants who were currently working, were younger than retirement age, had no more than 3 missing values in the MBI [2], and submitted plausible values for employment duration (no longer than age minus 14 years). This resulted in a final sample size of 27,020 (29.7%). The study was approved by the ethics committee of the Universitätsmedizin Halle under the registration number 2020-076.

Results from Model Tuning

eTable 1: Results from Model Tuning and Test Set rmse and R^2

Model	Tuning Parameters	Fixed Parameters	rmse	R^2
Linear Regression	None	None	0.662	0.584
Lasso Regression	penalty (λ) = 2.92 e-3	mixture = 1	0.661	0.585
Ridge Regression	penalty (λ) = 1.15 e-10	mixture = 0	0.662	0.584
Elastic Net Regression	penalty (λ) = 5.99 e-3;<br>mixture = 1	None	0.661	0.585
Random Forest	mtry = 17;<br>trees = 1111;<br>min_n = 2	None	0.665	0.583
XG-Boost	mtry = 12;<br>min_n = 14;<br>tree_depth = 10;<br>learn_rate = 1.29 e-2;<br>loss_reduction = 1 e-10;<br>sample_size = 0.1;<br>stop_iter = 12	trees = 1000	0.657<br>(0.6568)	0.590<br>(0.5904)
Multilayer Perceptron	hidden_units = 2;<br>penalty = 7.74 e-2;<br>epochs = 560	None	0.657<br>(0.6571)	0.590<br>(0.5899)
Multilayer Perceptron Ensemble	hidden_units = 7;<br>penalty = 1;<br>epochs = 340	None	0.658	0.589

Full Variable List

eTable 2: Sample Descriptive Statistics for all Variables

Variable	*Valid*	*NA (%)*	*Type*	*Mean**	*Median**	*SD**	*Range**
State	27020	0	factor	15 levels			
Professional Field	26839	0.7	factor	21 levels			
Occupational Area	24778	8.3	factor	10 levels			
Occupational Main Group	24170	10.5	factor	37 levels			
Occupational Group	23650	12.5	factor	150 levels			
Employment Duration (Years)	27020	0	numeric	17.32	15	12.22	0-53
Employment Type	26839	0.7	factor	Employed with Permanent Contract, Employed with Fixed-Term Contract, Civil-Servant, Temporary Work, Self-Employed			
Contractually Agreed Weekly Working Hours	26616	1.5	numeric	34.62	38.5	8.43	0-60
Actual Weekly Working Hours	26775	0.9	numeric	38.15	40	9.87	0-80
Personal Contact with Clients	26883	0.5	scale	1.96	2	1.16	0-3
Work at (Computer) Screen	26997	0.1	scale	1.97	2	0.98	0-3
Leadership Position	26295	2.7	dummy	0.25			
Ever Worked from Home	26933	0.3	dummy	0.56			
Currently Working from Home	26887	0.5	dummy	0.31			
Hours spent Working from Home	26866	0.6	numeric	5.86	0	10.81	0-80

Variable	*Valid*	*NA (%)*	*Type*	*Mean**	*Median**	*SD**	*Range**
Work-Life-Interference	26971	0.2	scale	2.26	2	1.12	1-5
Subjective Mental Health	26651	1.4	scale	3.38	3	0.95	1-5
Number of Household Members	26936	0.3	numeric	2.52	2	1.15	1-7
Gender	26971	0.2	factor	Male, Female, Diverse			
Age (Years)	27020	0	numeric	46.80	49	11.16	18-67
Country of Birth	26976	0.2	factor	Germany, Other Country in Europe, Country Outside Europe			
Continent of Birth	26975	0.2	factor	6 levels			
Age of Arrival in Germany	26972	0.2	numeric	0.44	0	3.48	0-63
Mother born in Germany	26965	0.2	dummy	0.89			
Father born in Germany	26792	0.8	dummy	0.88			
Mother's Continent of Birth	26955	0.2	factor	6 levels			
Father's Continent of Birth	26781	0.9	factor	6 levels			
Marital Status	26684	1.2	factor	Married, Not Married, Divorced, Widowed, Married but Separated from Partner			
Partnership	26682	1.3	dummy	0.81			
School Leaving Qualification	26743	1.0	scale	2.64	3	0.53	0-3
Monthly Household Net Income	24981	7.5	scale	5.13	5	1.62	1-7
Shift Work	26854	0.6	dummy	0.14			
Working Overtime	26494	1.9	dummy	0.66			

Variable	*Valid*	*NA (%)*	*Type*	*Mean**	*Median**	*SD**	*Range**
Occupation	26601	1.6	factor	2971 levels			
Burnout	27020	0	16 Item mean	1.74	1.56	1.03	0-6
Anxiety	26655	1.4	7 Item mean	0.82	0.71	0.60	0-3
Depression	26636	1.4	9 Item mean	0.67	0.56	0.50	0-3
Effort-Reward-Imbalance	25621	5.2	10 Item mean	2.55	2.50	0.51	1-4
Overcommitment	26510	1.9	6 Item mean	2.49	2.50	0.64	1-4
Resilience	26650	1.4	2 Item mean	3.15	3	0.69	0-4
Pain	26483	2.0	3 Item mean	0.68	0.67	0.51	0-2
Life Satisfaction	26624	1.5	3 Item mean	7.11	7.67	1.73	0-10
Number of Children in Household	26936	0.3	numeric	0.63	0	0.90	0-5
Education	26681	1.3	scale	1.91	1	1.08	0-4
Night Shift	26854	0.6	dummy	0.05			
Early Shift	26854	0.6	dummy	0.09			
Late Shift	26854	0.6	dummy	0.11			
On-Call Duty	26854	0.6	dummy	0.03			
Day Shift	26854	0.6	dummy	0.06			
Other Shift (e.g. 24-Hour-Shift)	26854	0.6	dummy	0.02			
More than one Employment	27020	0	dummy	0.05			

Note. Factor variables with more than 15 levels were converted to likelihood encodings prior to machine learning analysis. * = not available for factors. For type dummy, 0 means no and 1 means yes.

Effect Sizes for all Variables

eTable 3: Simple Linear Associations with Burnout

Variable	*Correlation*	R^2
Burnout: Depersonalisation	0.867	0.751
Burnout: Emotional Exhaustion	0.820	0.673
Burnout: Reduced Personal Achievement	0.659	0.434
Depression	0.638	0.407
Anxiety	0.558	0.311
Life Satisfaction	-0.553	0.306
Subjective Mental Health	-0.543	0.295
Effort-Reward-Imbalance	0.524	0.274
Overcommitment	0.464	0.215
Work-Life-Interference	0.450	0.202
Resilience	-0.396	0.157
Pain	0.308	0.095
Occupation*	*NA*	0.022
Employment Type*	*NA*	0.015
Leadership Position	-0.109	0.012
Occupational Group*	*NA*	0.011
Marital Status*	*NA*	0.009
Age	-0.096	0.009
Monthly Household Net Income	-0.079	0.006
Professional Field*	*NA*	0.005
Working Overtime	0.068	0.005
Occupational Main Group*	*NA*	0.004
Early Shift	0.059	0.004

Variable	*Correlation*	*R²*
Personal Contact with Clients	-0.058	0.003
Number of Household Members	-0.055	0.003
Shift Work	0.055	0.003
Partnership	-0.055	0.003
Late Shift	0.052	0.003
Federal District*	*NA*	0.003
Number of Children in Household	-0.050	0.002
Gender*	*NA*	0.002
Actual Weekly Working Hours	0.046	0.002
Work at (Computer) Screen	0.044	0.002
Night Shift	0.043	0.002
State*	*NA*	0.002
Employment Duration	-0.037	0.001
Day Shift	0.034	0.001
Contractually Agreed Weekly Working Hours	0.032	0.001
Father's Continent of Birth*	*NA*	0.001
Mother's Continent of Birth*	*NA*	0.000
More than one Employment	-0.021	0.000
Education	-0.019	0.000
Other Shift	0.019	0.000
Ever worked from Home	0.014	0.000
Continent of Birth*	*NA*	0.000
On-Call Duty	0.011	0.000
Country of Birth*	*NA*	0.000
Age of Arrival in Germany	0.008	0.000
Currently Working from Home	0.007	0.000

Variable	*Correlation*	*R^2*
Hours Spent Working from Home	0.005	0.000
Mother born in Germany	-0.004	0.000
School Leaving Qualification	0.001	0.000
Father born in Germany	0.001	0.000

Note. Simple linear associations with burnout with R^2 for all variables, ordered by R^2. NA = not applicable for factor variables marked with an asterisk (*), Correlation = Pearson correlations.

Association of Burnout with Occupation and Occupational Group

eFigure 1: Wordcloud for the Association of Burnout with Occupation

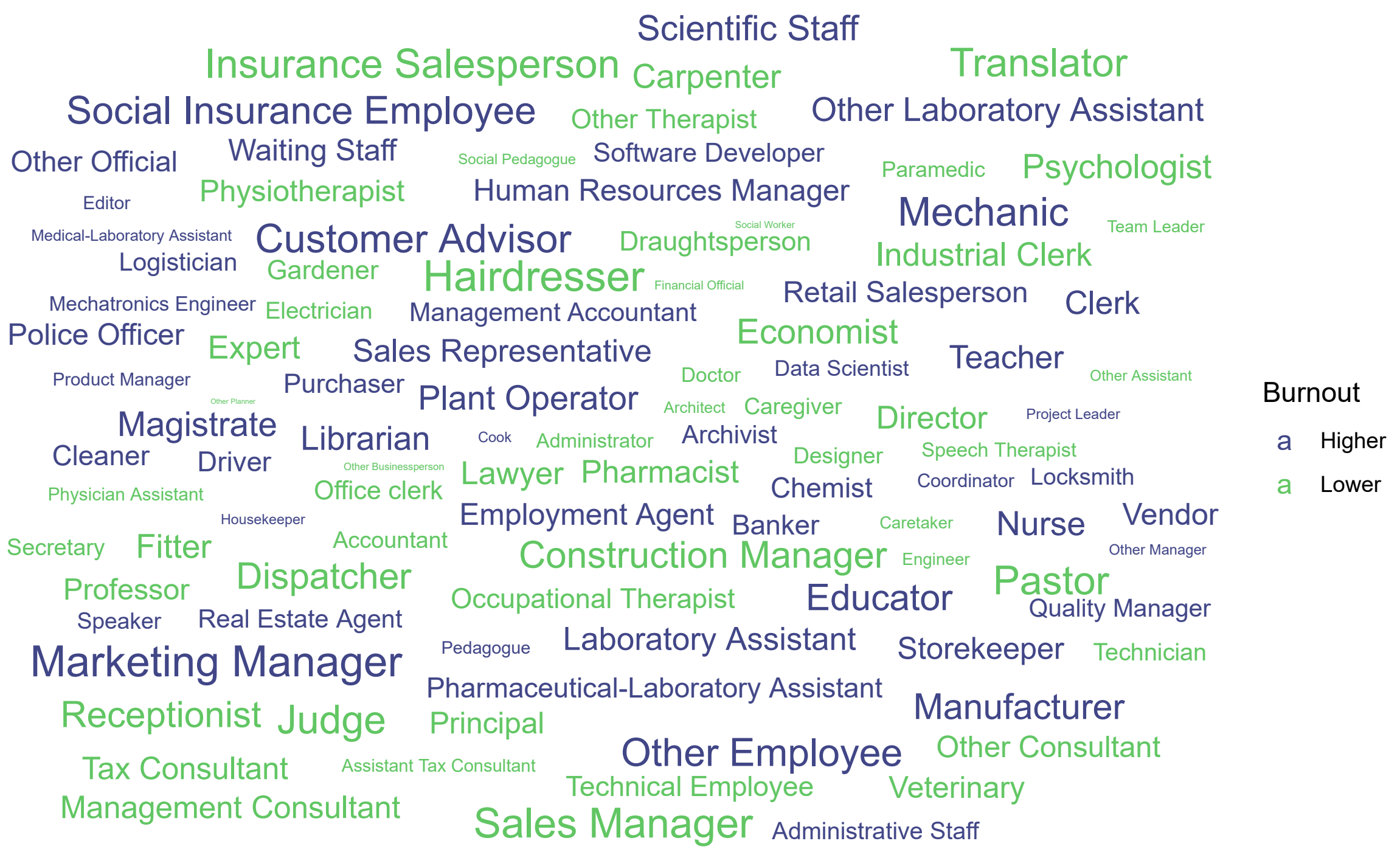

Note. Larger words mean the influence on burnout is higher and colour represents the direction of association: green (light) signifies a lower burnout mean in this occupation and blue (dark) signifies a higher mean. The occupation means were calculated if there were at least 30 participants in the group. The absolute z-standardised values determine word size, which thus would be zero at the unweighted burnout mean of the occupations.

eFigure 2: Wordcloud for the Association of Burnout with Occupational Group

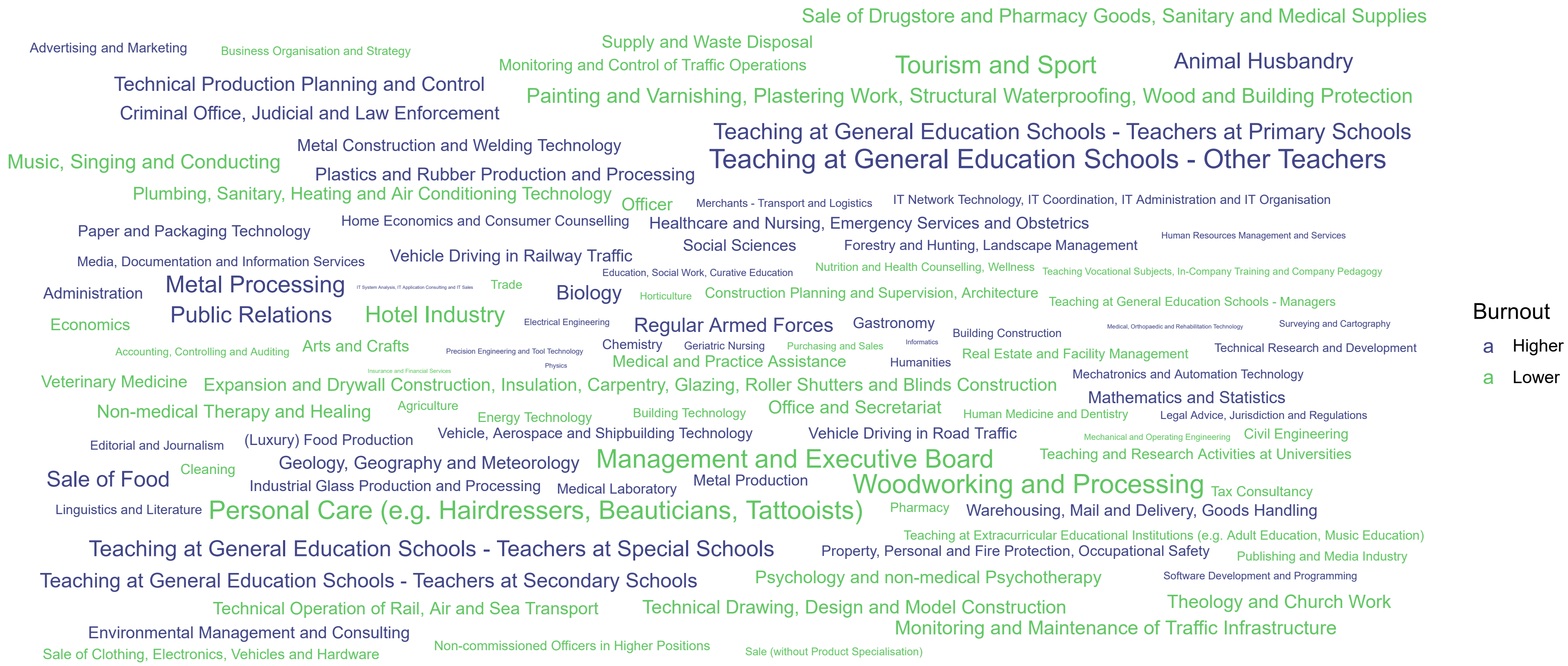

Note. Larger words mean the influence on burnout is higher and colour represents the direction of association: green (light) signifies a lower burnout mean in this occupational group and blue (dark) signifies a higher mean. The occupational group means were calculated if there were at least 30 participants in the group. The absolute z-standardised values determine word size, which thus would be zero at the unweighted burnout mean of the occupational groups.

eTable 4: Mean Burnout Values by Occupation

Occupation	*Mean Burnout*	*z*
Marketing Manager	2.089	2.257
Mechanic	2.023	1.863
Customer Advisor	2.021	1.852
Other Employee	2.006	1.762
Social Insurance Employee	2.002	1.741
Educator	1.965	1.524
Manufacturer	1.950	1.436
Scientific Staff	1.938	1.361
Magistrate	1.924	1.282
Plant Operator	1.917	1.242
Nurse	1.912	1.212
Librarian	1.910	1.197
Other Laboratory Assistant	1.905	1.171
Clerk	1.895	1.108
Teacher	1.894	1.107
Laboratory Assistant	1.891	1.089
Sales Representative	1.882	1.033
Storekeeper	1.871	0.967
Vendor	1.866	0.940
Employment Agent	1.864	0.930
Human Resources Manager	1.863	0.922
Waiting Staff	1.862	0.914
Police Officer	1.859	0.899
Retail Salesperson	1.851	0.851
Other Official	1.845	0.817

Occupation	*Mean Burnout*	*z*
Pharmaceutical-Laboratory Assistant	1.835	0.756
Cleaner	1.833	0.747
Chemist	1.833	0.745
Driver	1.832	0.741
Banker	1.832	0.737
Purchaser	1.819	0.663
Software Developer	1.817	0.649
Management Accountant	1.811	0.617
Real Estate Agent	1.806	0.585
Archivist	1.806	0.584
Logistician	1.803	0.567
Quality Manager	1.800	0.550
Administrative Staff	1.792	0.505
Locksmith	1.784	0.458
Speaker	1.779	0.427
Data Scientist	1.772	0.383
Mechatronics Engineer	1.759	0.307
Coordinator	1.748	0.241
Editor	1.744	0.223
Product Manager	1.743	0.216
Pedagogue	1.740	0.196
Medical-Laboratory Assistant	1.730	0.137
Other Manager	1.725	0.107
Cook	1.724	0.105
Project Leader	1.724	0.105
Housekeeper	1.724	0.100

Occupation	*Mean Burnout*	*z*
Other Planner	1.704	-0.015
Social Worker	1.702	-0.026
Other Businessperson	1.701	-0.031
Financial Official	1.696	-0.061
Social Pedagogue	1.688	-0.108
Other Assistant	1.687	-0.119
Architect	1.682	-0.144
Team Leader	1.678	-0.171
Caretaker	1.677	-0.173
Engineer	1.677	-0.177
Assistant Tax Consultant	1.671	-0.207
Physician Assistant	1.669	-0.220
Speech Therapist	1.658	-0.289
Administrator	1.656	-0.296
Doctor	1.654	-0.309
Paramedic	1.642	-0.379
Designer	1.636	-0.419
Caregiver	1.635	-0.424
Secretary	1.631	-0.445
Accountant	1.631	-0.446
Electrician	1.628	-0.466
Technician	1.620	-0.508
Office clerk	1.601	-0.625
Gardener	1.596	-0.654
Other Therapist	1.592	-0.675
Draughtsperson	1.589	-0.694

Occupation	*Mean Burnout*	*z*
Occupational Therapist	1.581	-0.740
Technical Employee	1.578	-0.756
Professor	1.559	-0.870
Other Consultant	1.556	-0.889
Physiotherapist	1.552	-0.912
Principal	1.551	-0.919
Veterinary	1.548	-0.933
Tax Consultant	1.535	-1.014
Management Consultant	1.534	-1.018
Pharmacist	1.529	-1.045
Director	1.526	-1.063
Lawyer	1.526	-1.066
Expert	1.523	-1.082
Industrial Clerk	1.516	-1.123
Fitter	1.506	-1.185
Carpenter	1.501	-1.210
Psychologist	1.497	-1.238
Economist	1.484	-1.314
Receptionist	1.453	-1.497
Dispatcher	1.449	-1.519
Construction Manager	1.436	-1.595
Translator	1.391	-1.859
Judge	1.387	-1.884
Pastor	1.385	-1.895
Insurance Salesperson	1.377	-1.946
Sales Manager	1.358	-2.056

Occupation	*Mean Burnout*	*z*
Hairdresser	1.331	-2.216

Note. Mean burnout values were calculated for every occupation present in the data at least 30 times. z = z-standardised values.

eTable 5: Mean Burnout Values by Occupational Groups

Occupational Group	*mean*	*z*
Teaching at General Education Schools - Other Teachers	2.179	2.842
Metal Processing	2.049	2.045
Public Relations	2.020	1.866
Teaching at General Education Schools - Teachers at Primary Schools	2.014	1.832
Teaching at General Education Schools - Teachers at Special Schools	2.004	1.770
Animal Husbandry	1.999	1.736
Sale of Food	1.993	1.703
Teaching at General Education Schools - Teachers at Secondary Schools	1.946	1.413
Biology	1.939	1.371
Regular Armed Forces	1.933	1.333
Technical Production Planning and Control	1.930	1.314
Criminal Office, Judicial and Law Enforcement	1.899	1.128
Plastics and Rubber Production and Processing	1.883	1.029
Geology, Geography and Meteorology	1.881	1.013
Vehicle Driving in Railway Traffic	1.856	0.860
Metal Construction and Welding Technology	1.852	0.840
Mathematics and Statistics	1.852	0.837
Environmental Management and Consulting	1.850	0.828
Healthcare and Nursing, Emergency Services and Obstetrics	1.846	0.800
Warehousing, Mail and Delivery, Goods Handling	1.842	0.776

Occupational Group	*mean*	*z*
Social Sciences	1.840	0.764
Administration	1.833	0.720
Paper and Packaging Technology	1.830	0.703
Vehicle Driving in Road Traffic	1.830	0.703
Metal Production	1.825	0.670
Industrial Glass Production and Processing	1.823	0.659
Gastronomy	1.819	0.635
(Luxury) Food Production	1.818	0.631
Property, Personal and Fire Protection, Occupational Safety	1.812	0.593
Vehicle, Aerospace and Shipbuilding Technology	1.810	0.582
Home Economics and Consumer Counselling	1.805	0.547
Medical Laboratory	1.801	0.528
Forestry and Hunting, Landscape Management	1.800	0.520
Advertising and Marketing	1.793	0.474
Media, Documentation and Information Services	1.791	0.463
Chemistry	1.789	0.451
Linguistics and Literature	1.785	0.430
IT Network Technology, IT Coordination, IT Administration and IT Organisation	1.778	0.382
Editorial and Journalism	1.776	0.373
Mechatronics and Automation Technology	1.775	0.363
Humanities	1.770	0.338
Technical Research and Development	1.768	0.323
Building Construction	1.762	0.286
Merchants - Transport and Logistics	1.756	0.247
Legal Advice, Jurisdiction and Regulations	1.755	0.245
Geriatric Nursing	1.752	0.227

Occupational Group	*mean*	*z*
Software Development and Programming	1.750	0.215
Education, Social Work, Curative Education	1.743	0.168
Surveying and Cartography	1.736	0.126
Human Resources Management and Services	1.735	0.120
Electrical Engineering	1.734	0.113
Precision Engineering and Tool Technology	1.722	0.044
Informatics	1.720	0.030
Physics	1.719	0.024
Medical, Orthopaedic and Rehabilitation Technology	1.718	0.014
IT System Analysis, IT Application Consulting and IT Sales	1.716	0.003
Insurance and Financial Services	1.713	-0.011
Mechanical and Operating Engineering	1.700	-0.093
Purchasing and Sales	1.688	-0.170
Teaching Vocational Subjects, In-Company Training and Company Pedagogy	1.687	-0.173
Horticulture	1.685	-0.183
Accounting, Controlling and Auditing	1.678	-0.230
Sale (without Product Specialisation)	1.677	-0.234
Nutrition and Health Counselling, Wellness	1.668	-0.289
Business Organisation and Strategy	1.664	-0.316
Human Medicine and Dentistry	1.662	-0.328
Trade	1.658	-0.352
Teaching at General Education Schools - Managers	1.654	-0.373
Agriculture	1.653	-0.383
Building Technology	1.652	-0.385
Publishing and Media Industry	1.652	-0.388

Occupational Group	*mean*	*z*
Teaching at Extracurricular Educational Institutions (e.g. Adult Education, Music Education)	1.648	-0.410
Pharmacy	1.645	-0.430
Non-commissioned Officers in Higher Positions	1.644	-0.438
Energy Technology	1.639	-0.465
Real Estate and Facility Management	1.638	-0.474
Tax Consultancy	1.633	-0.502
Cleaning	1.632	-0.508
Civil Engineering	1.632	-0.511
Sale of Clothing, Electronics, Vehicles and Hardware	1.624	-0.562
Construction Planning and Supervision, Architecture	1.623	-0.565
Teaching and Research Activities at Universities	1.619	-0.590
Monitoring and Control of Traffic Operations	1.595	-0.739
Arts and Crafts	1.591	-0.761
Medical and Practice Assistance	1.587	-0.788
Economics	1.584	-0.804
Veterinary Medicine	1.577	-0.846
Supply and Waste Disposal	1.562	-0.936
Technical Operation of Rail, Air and Sea Transport	1.562	-0.936
Officer	1.558	-0.964
Psychology and non-medical Psychotherapy	1.555	-0.979
Plumbing, Sanitary, Heating and Air Conditioning Technology	1.552	-1.003
Expansion and Drywall Construction, Insulation, Carpentry, Glazing, Roller Shutters and Blinds Construction	1.548	-1.024
Office and Secretariat	1.547	-1.029
Non-medical Therapy and Healing	1.543	-1.054
Theology and Church Work	1.534	-1.110

Occupational Group	*mean*	*z*
Technical Drawing, Design and Model Construction	1.526	-1.162
Monitoring and Maintenance of Traffic Infrastructure	1.512	-1.245
Sale of Drugstore and Pharmacy Goods, Sanitary and Medical Supplies	1.501	-1.315
Music, Singing and Conducting	1.496	-1.340
Painting and Varnishing, Plastering Work, Structural Waterproofing, Wood and Building Protection	1.476	-1.467
Hotel Industry	1.406	-1.893
Tourism and Sport	1.331	-2.353
Personal Care (e.g. Hairdressers, Beauticians, Tattooists)	1.299	-2.550
Management and Executive Board	1.291	-2.601
Woodworking and Processing	1.260	-2.788

Note. Mean burnout values were calculated for every occupational group present in the data at least 30 times. z = z-standardised values.

Fractional Feature Contributions for all Variables

eFigure 3: Variable Importance Plot

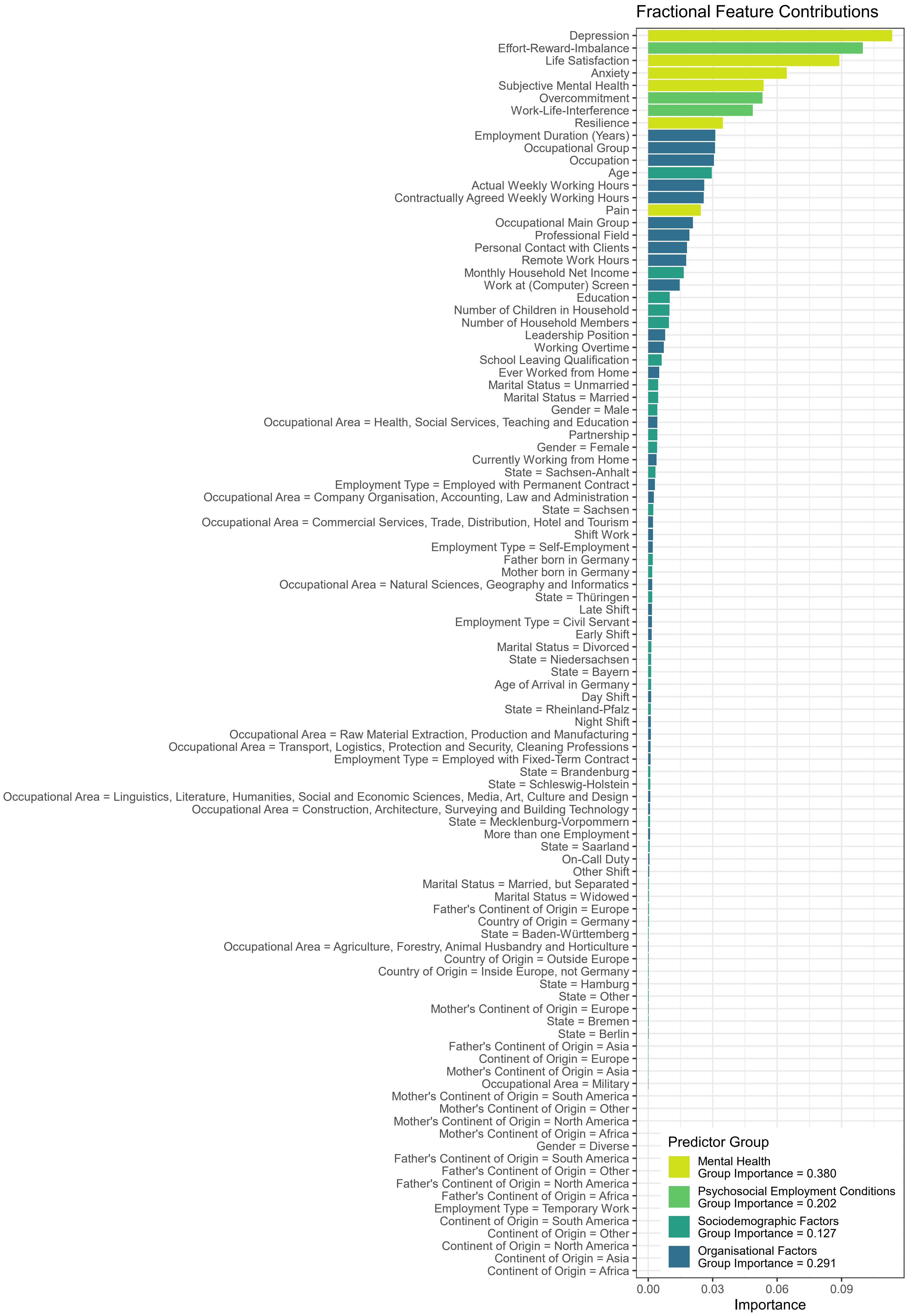

Note. Variable Importance scores from xg-boost model of all features of burnout, features are sorted by importance. Feature groups are colour coded, the group importance is the sum of fractional feature contributions of each variable in this group.

Beeswarm Plot for the 21 Most Important Features

eFigure 4: Beeswarm Plot

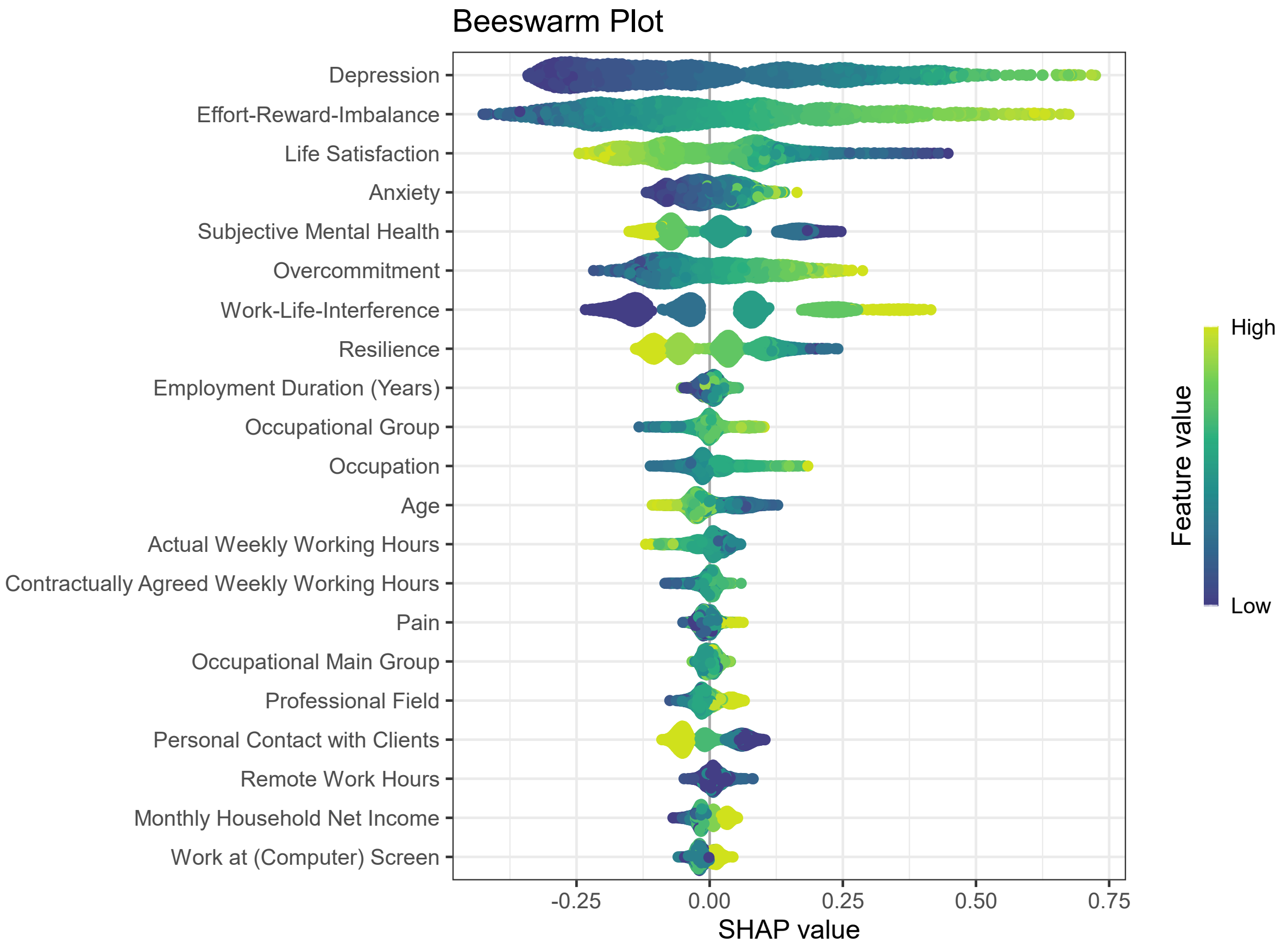

Note. Beeswarm plot for the most important features according to their fractional contribution, ordered by importance. Each point represents the SHAP value (x-axis) of the associated feature (row). The feature value is represented by colour: low values are darker (blue) and high values are lighter (yellow). A negative SHAP value means that the feature lowers the overall outcome (= lower burnout), positive SHAP values mean that the associated feature increases the outcome (= higher burnout). The greater the absolute SHAP value, the higher is the influence on the prediction. If the plot results in a colour gradient, it means that the association of this feature is close to linear. If the darker side of the gradient is on the left-hand side, the association is positive, e.g., higher values for burnout are predicted if depression is higher. If the lighter side is on the left-hand side, then the association is negative, e.g., higher values for burnout are predicted if the life satisfaction is lower.

SHAP Dependence Plots with Individual Y-Axis Scales

eFigure 5: SHAP Dependence Plots

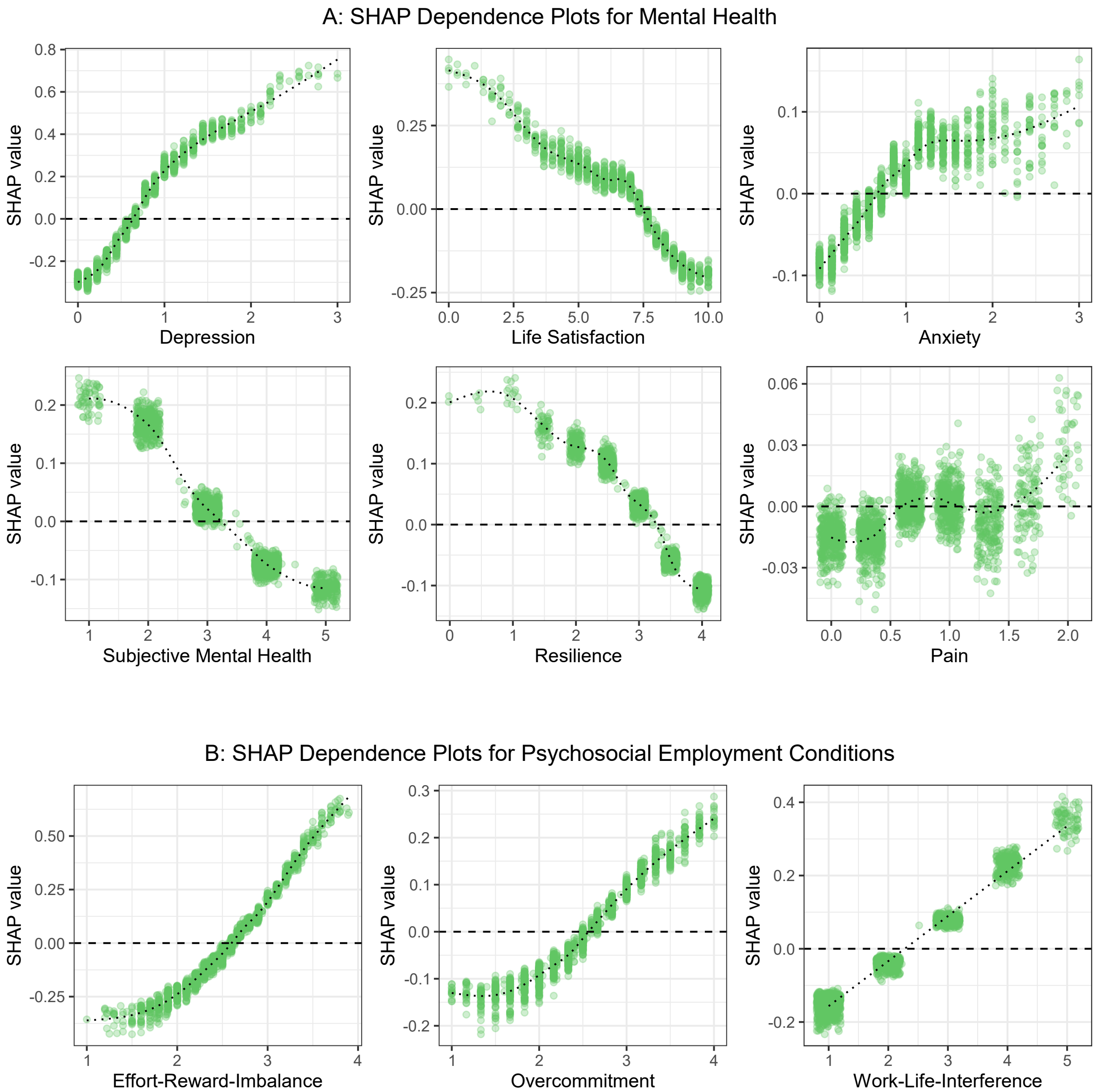

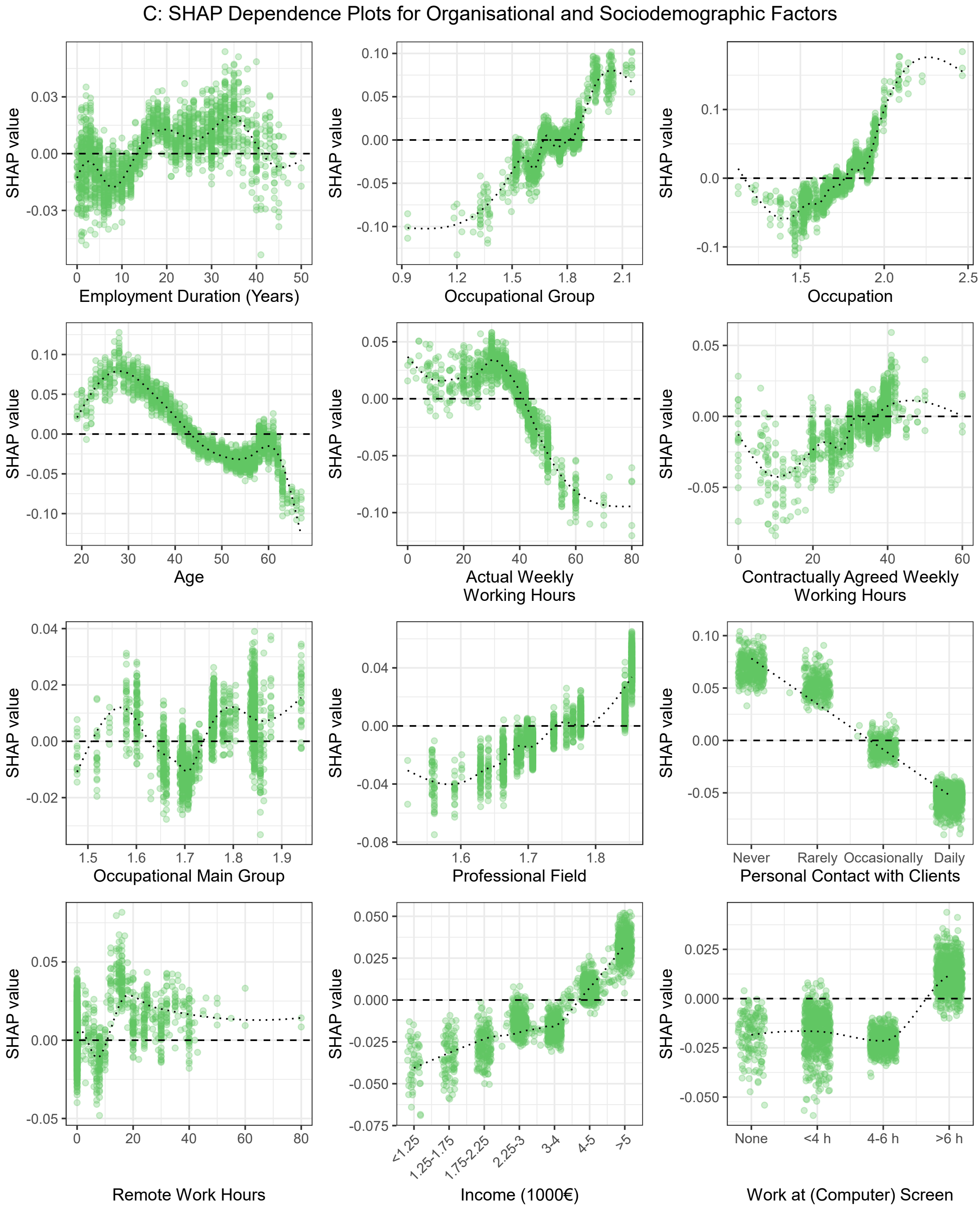
C: SHAP Dependence Plots for Organisational and Sociodemographic Factors
SHAP value
Employment Duration (Years)
Occupational Group
Occupation
Age
Actual Weekly Working Hours
Contractually Agreed Weekly Working Hours
Occupational Main Group
Professional Field
Personal Contact with Clients
Never
Rarely
Occasionally
Daily
Remote Work Hours
Income (1000€)
<1.25
1.25-1.75
1.75-2.25
2.25-3
3-4
4-5
>5
Work at (Computer) Screen
None
<4 h
4-6 h
>6 h

Model with Case Weights

Method

Importance weights were calculated for each participant to adjust the sample for age, gender and school leaving qualification. Population data was retrieved from the microcensus, which is a large annual household survey in Germany [3]. It contains the data on the frequency distribution of the population along the variables age in 5-year steps from 20 to 65 years × gender (male and female) × school leaving qualification. To determine the according distribution in the sample, we excluded participants outside the age and gender range as well as participants with missing values on at least one of those variables, which resulted in a sample size of 26,300 participants. Weights were calculated by dividing the population proportion by the sample proportion.

Results

The top 21 features were the same as in the unweighted model, although the cut in importance to the next feature is not clear in this model (eFigure 6). Some features have slightly different importance values and therefore changed their places in the ranking: life satisfaction and anxiety have changed places, resilience was more important and occupation was less important in the original model, pain and contractually agreed weekly working hours have changed places as well as hours spent in remote work and personal contact with clients. The prediction patterns were also very similar with the original model (eFigures 7 and 8).

Limitations

The largest limitation is that the sample was not weighted for migration background, and it only contains few participants who were not born in Germany. Another limitation is

that our sample did not include any woman between 20 to 24 years with a degree after 9th grade nor any woman between 45 to 49 years without school leaving qualification.

eFigure 6: Variable Importance Plot for Weighted Model

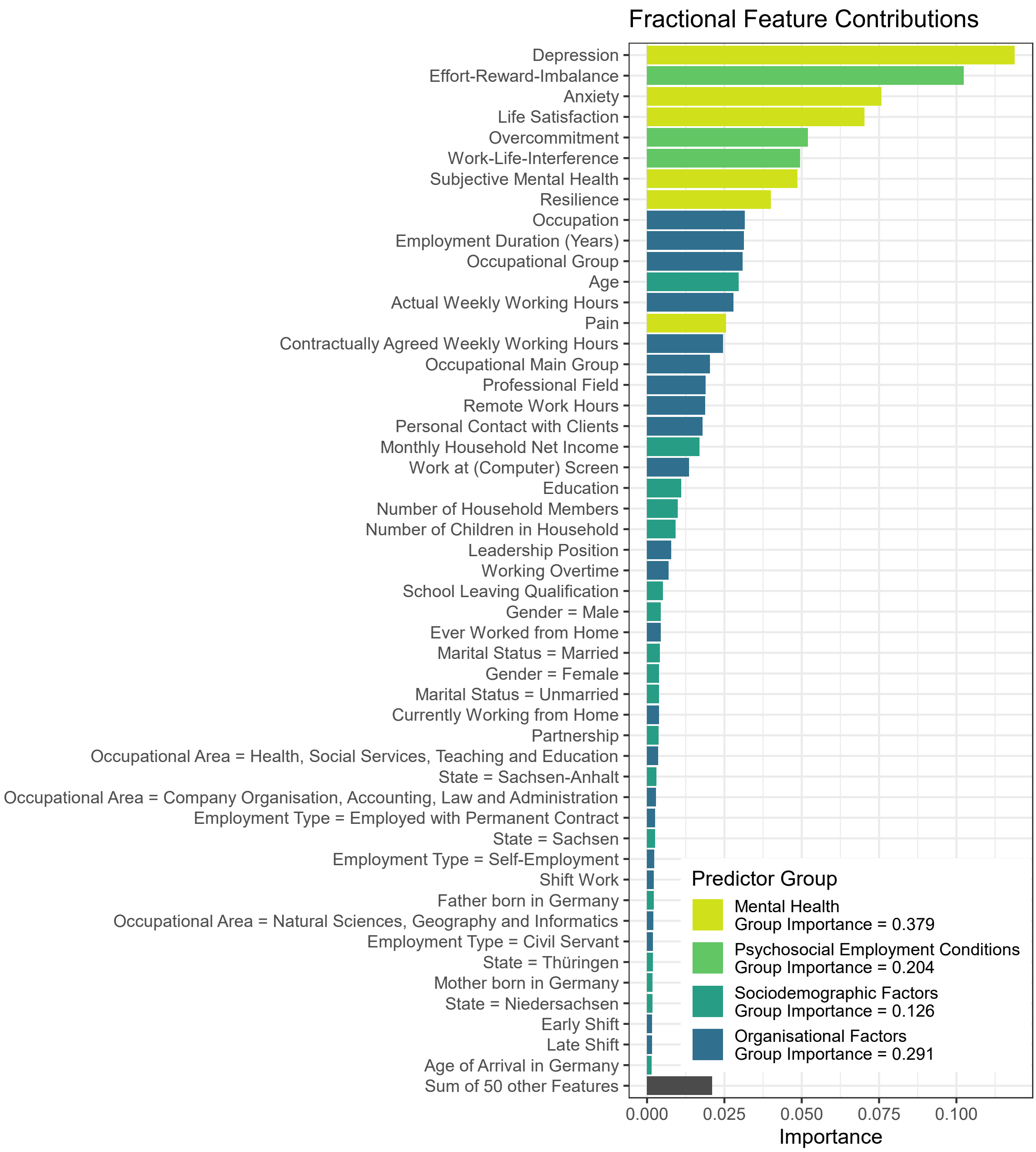

Note. Variable Importance scores for weighted xg-boost model, features are sorted by importance. Feature groups are colour coded, the group importance is the sum of fractional feature contributions of each variable in this group.

eFigure 7: Beeswarm Plot for Weighted Model

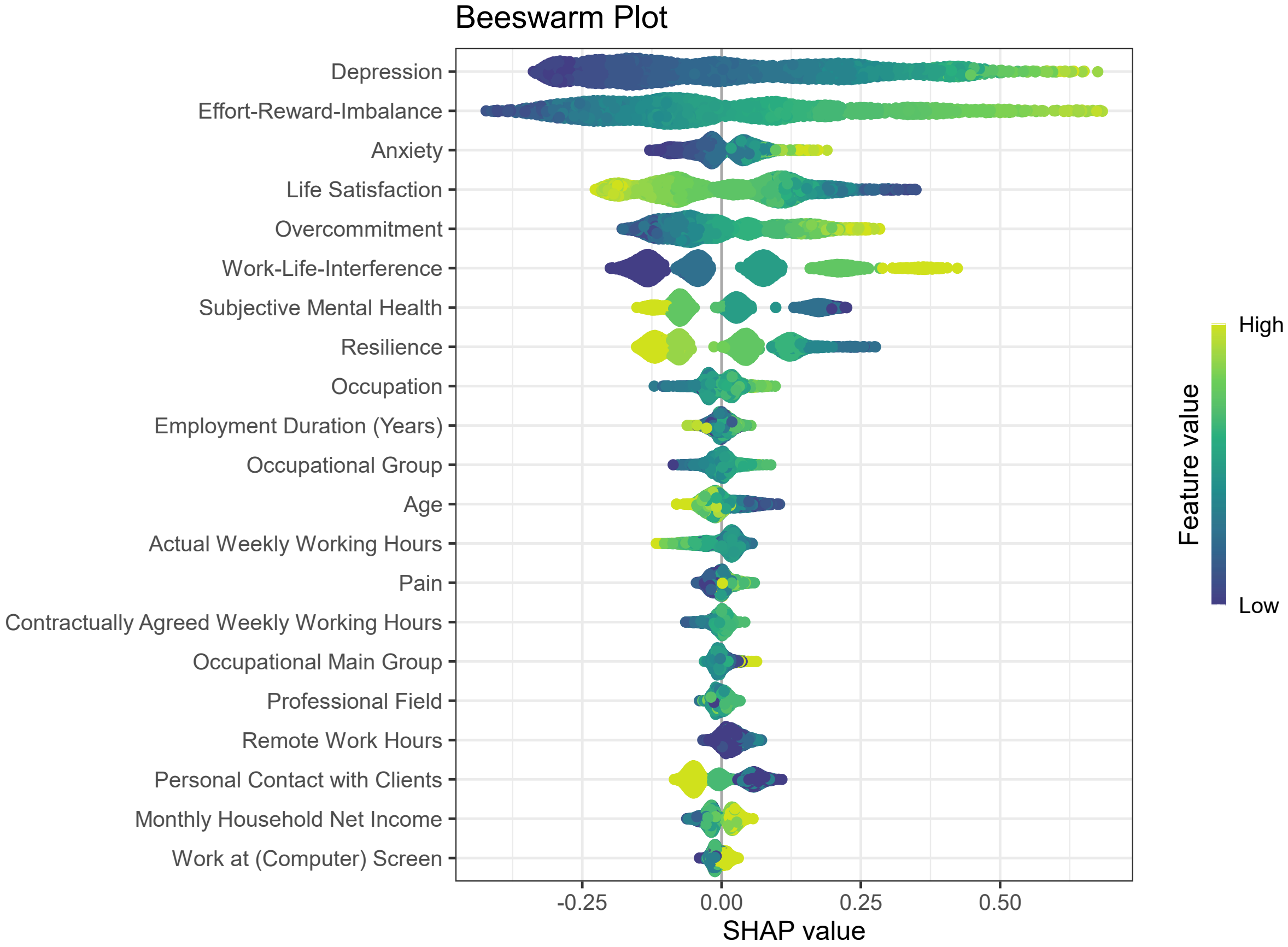

Note. Beeswarm plot for the most important features according to their fractional contribution, ordered by importance. Each point represents the SHAP value (x-axis) of the associated feature (row). The feature value is represented by colour: low values are darker (blue) and high values are lighter (yellow). A negative SHAP value means that the feature lowers the overall outcome (= lower burnout), positive SHAP values mean that the associated feature increases the outcome (= higher burnout). The greater the absolute SHAP value, the higher is the influence on the prediction. If the plot results in a colour gradient, it means that the association of this feature is close to linear. If the darker side of the gradient is on the left-hand side, the association is positive, e.g., higher values for burnout are predicted if depression is higher. If the lighter side is on the left-hand side, then the association is negative, e.g., higher values for burnout are predicted if the life satisfaction is lower.

eFigure 8: SHAP Dependence Plots for Weighted Model

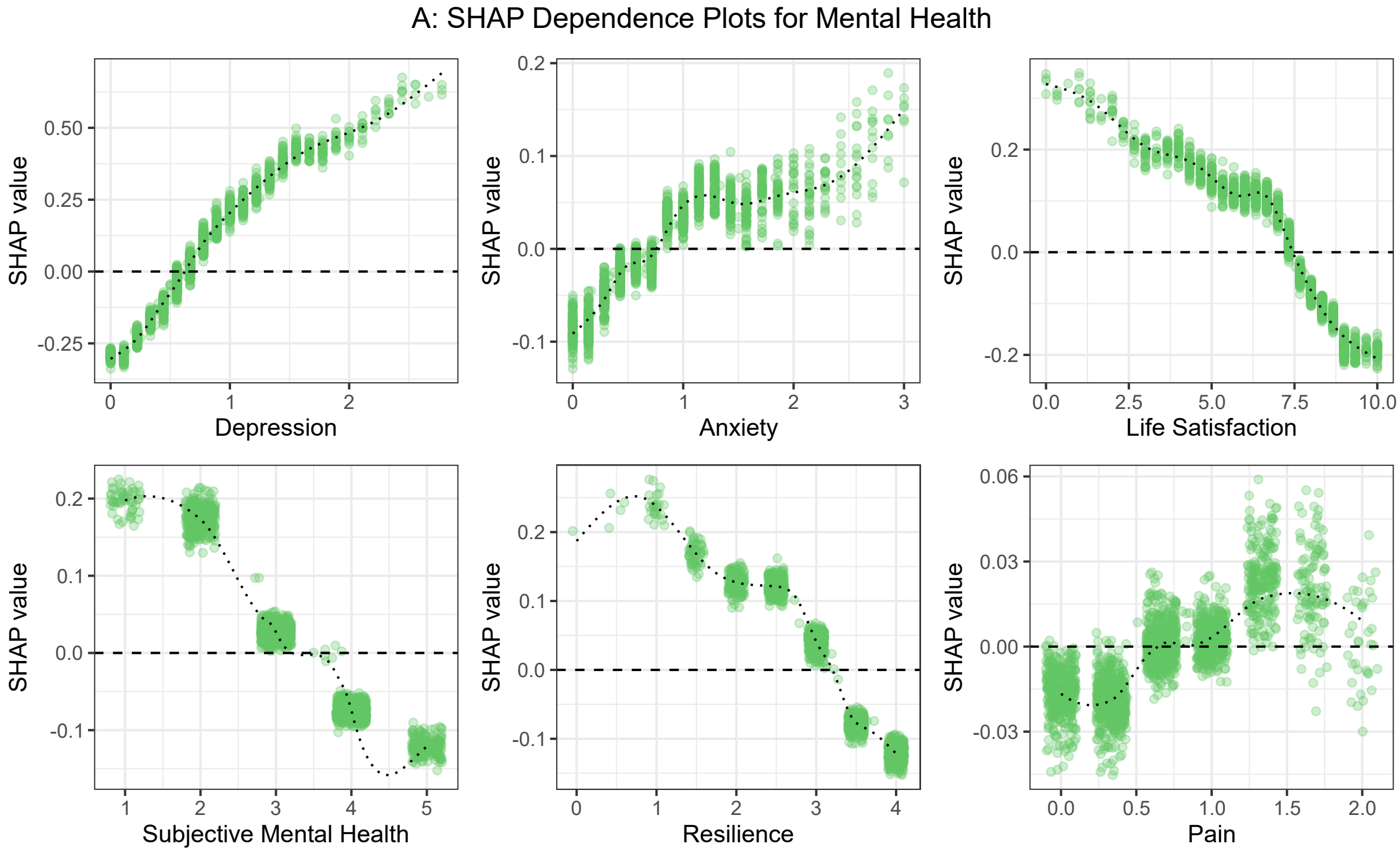

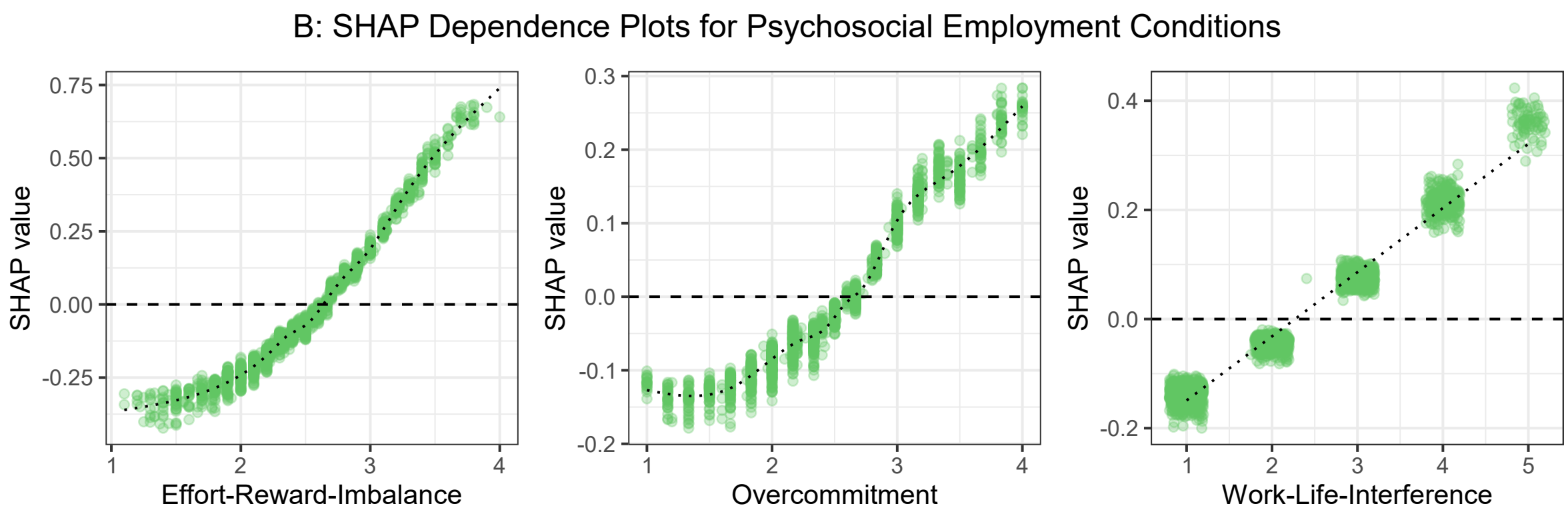

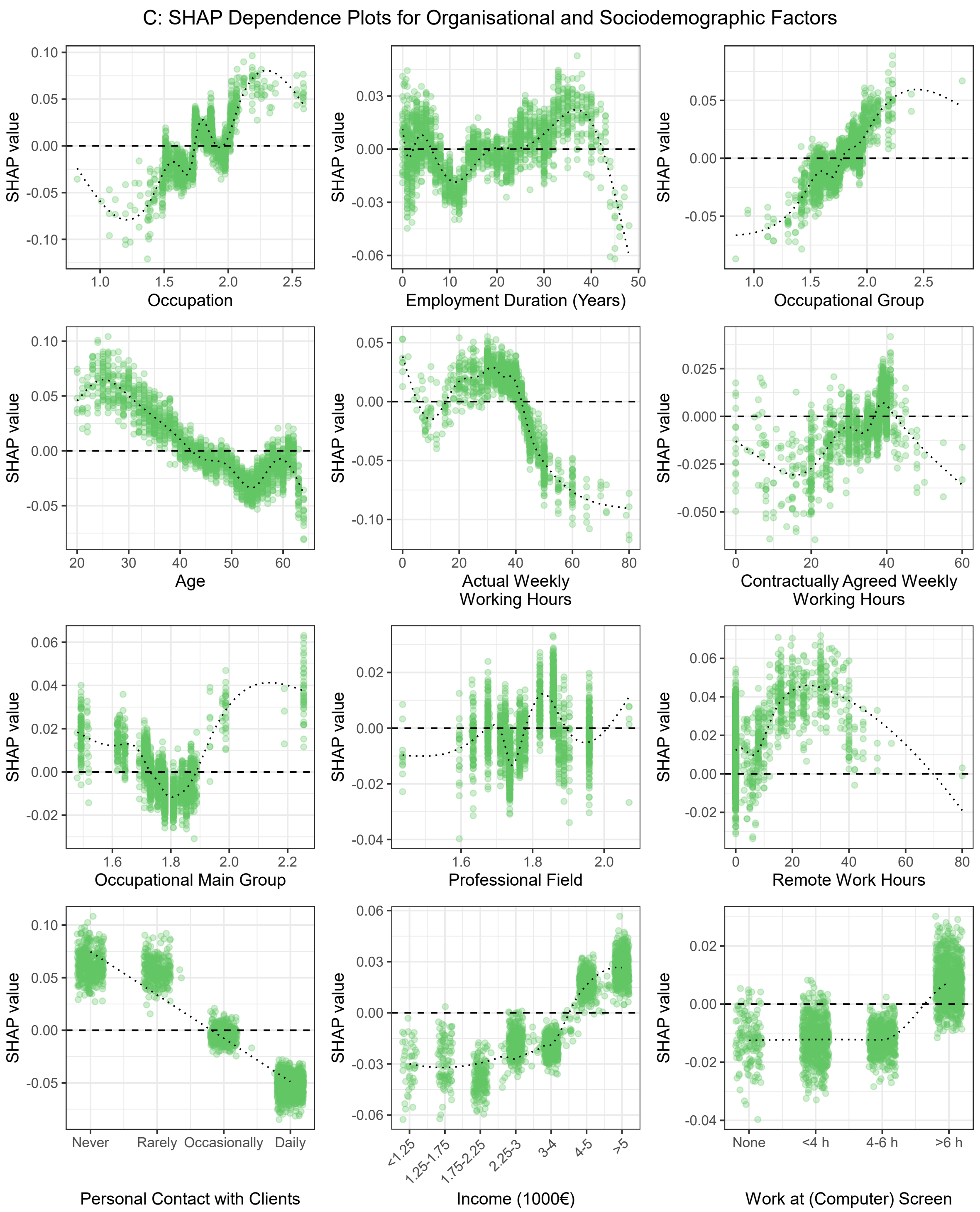
C: SHAP Dependence Plots for Organisational and Sociodemographic Factors
SHAP value
Occupation
Employment Duration (Years)
Occupational Group
Age
Actual Weekly Working Hours
Contractually Agreed Weekly Working Hours
Occupational Main Group
Professional Field
Remote Work Hours
Never
Rarely
Occasionally
Daily
Personal Contact with Clients
<1.25
1.25-1.75
1.75-2.25
2.25-3
3-4
4-5
>5
Income (1000€)
None
<4 h
4-6 h
>6 h
Work at (Computer) Screen

References

1. Broda A, Klee B, Diexer S et al. (2024) DigiHero – Populationsbasierte Kohortenstudie für digitale Gesundheitsforschung in Deutschland. Ärzteblatt Sachsen-Anhalt 0102/2024
2. Schaufeli W, Leiter M, Maslach C et al. (1996) Maslach Burnout Inventory - General Survey (GS). Maslach Burnout Inventory Manual 31
3. Statistisches Bundesamt (2025) Bevölkerung (ab 15 Jahren): Deutschland, Jahre (bis 2019), Geschlecht, Altersgruppen, Allgemeine Schulausbildung. Code: 12211-9012. https://www-genesis.destatis.de/datenbank/online/table/12211-9012/table-toolbar. Accessed 25 Aug 2025
